# Multi-tissue resource characteristics and comprehensive utilization of medicinal and edible loquat (*Eriobotrya japonica*): a review

**DOI:** 10.3389/fpls.2026.1953326

**Published:** 2026-10-05

**Authors:** Xinyue Wang, Shuzhi Tang, Hailan Lin, Bisha Wu, Shunquan Lin, Jincheng Wu, Shoukai Lin

**Affiliations:** 1 College of Environmental and Biological Engineering, Putian University, Putian, Fujian, China; 2 Fujian Provincial Key Laboratory of Ecological Impacts and Treatment Technologies for Emerging Contaminants, Putian University, Putian, Fujian, China; 3 Key Laboratory of Ecological Environment and Information Atlas, Putian University, Putian, Fujian, China; 4 State Key Laboratory for Conservation and Utilization of Subtropical Agro-bioresources, College of Horticulture, South China Agricultural University, Guangzhou, Guangdong, China

**Keywords:** anti-inflammatory, antioxidant, bioactive composition, food application, loquat (*Eriobotrya japonica*), triterpenoid compounds

## Abstract

Loquat (*Eriobotrya japonica* (Thunb.) Lindl.), a member of the Rosaceae family, exhibits pronounced tissue-specific metabolic specialization that underpins its nutritional value, pharmacological activities, and industrial potential. Contemporary research on loquat has largely focused on single tissues or individual bioactive compounds, whereas comprehensive cross-tissue comparative profiling and mechanistic analyses remain substantially underexplored. This fragmented research paradigm obscures both the tissue-specific distribution of secondary metabolites and the upstream regulatory networks governing their biosynthesis, resulting in the inefficient exploitation of by-products such as peels and seeds. In this review, we synthesize several decades of empirical and mechanistic studies on the phytochemical profiles, pharmacological functions, and resource-transformation mechanisms of the core bioactive constituents across five major loquat tissues—pulp, peel, seed, leaf, and flower. We clarify the core metabolites conserved across all tissues and the signature compounds specific to individual organs, and summarize their pleiotropic biological activities, including antioxidant, anti-inflammatory, antitumor, hypoglycemic, and multi-organ cytoprotective effects. Beyond phenotypic description, we further examine the evolutionary genomic origins and hierarchical regulatory networks underlying tissue-specific metabolism: lineage-specific whole-genome duplication is proposed to contribute to the heterogeneous accumulation of triterpenoids across tissues, whereas cold-adaptive signaling and developmental hormonal modulation cooperatively reshape organ-specific secondary metabolic reprogramming. Finally, we identify three pivotal industrial constraints on the sustainable valorization of loquat resources—fragmented processing layouts, low-efficiency recycling of agricultural residual biomass, and frost-induced instability of raw material supply—and propose targeted transformation strategies, including marker-assisted germplasm improvement, multi-omics dissection of tissue-specific metabolic regulatory networks, synthetic-biology-based green biosynthesis of high-value phytochemicals, and the development of a comprehensive circular industrial system. These strategies provide a theoretical basis for the integrated utilization of the medicinal and edible resources of loquat.

## 1 Introduction

Loquat (*Eriobotrya japonica*), a subtropical evergreen perennial fruit tree of the Rosaceae family and an endemic food-medicine homologous species in China, has a documented cultivation history spanning more than 2000 years (Tian et al., 2011). Currently, its cultivation and commercial applications have expanded to over 30 countries and regions, including Spain, Japan, Italy, and the United States (Cai et al., 2019). Population genomic analysis based on RAD-tag resequencing and single-nucleotide polymorphism (SNP) markers has confirmed that modern loquat cultivars originated from a single domestication event with a non-significant genetic bottleneck, laying a genetic foundation for the rich diversity of bioactive compounds in different germplasms (Wang et al., 2017; Nagano et al., 2022; Jing et al., 2023). As a representative medicinal and edible homologous plant, loquat has been officially recorded in herbal classics for clearing lung heat, relieving cough, moistening intestine, and promoting fluid production (Zhao, 2024; Syahputra et al., 2025). In addition to edible pulp, leaves and flowers are new food raw materials recognized by the Chinese government and were approved and listed in the Catalogue of New Food Raw Materials promulgated by the National Health Commission of the People’s Republic of China (NHC) in 2014 and 2019, respectively (Announcement No. 20 of 2014; Announcement No. 2 of 2019).

Like many fruit trees, loquat exhibits pronounced tissue-specific metabolic specialization, with each organ—pulp, peel, seed, leaf, and flower—accumulating a characteristic repertoire of bioactive metabolites that underpins its nutritional value, pharmacological activities, and industrial potential. Advances in phytochemistry and pharmacology have enabled comprehensive identification and mechanistic validation of diverse bioactive metabolites across distinct loquat tissues, including triterpenoids (Xu et al., 2012; Yang et al., 2025), flavonoids (Hu et al., 2008), polyphenols (Song et al., 2010), volatile essential oils (Lu and Liu, 2008), and polysaccharides (Sagar et al., 2020; Zhang et al., 2021). These metabolites confer a broad spectrum of biological functions, including anti-inflammatory (Ge et al., 2009; Cha et al., 2011), antioxidant (Song et al., 2010), antiviral (De Tommasi et al., 1992), antitumor (Ito et al., 2002; Uto et al., 2013), hypoglycemic (Li et al., 2007; Lü et al., 2009; Wu et al., 2021b), and hypolipidemic activities (Shih et al., 2010; Khouya et al., 2022). Meanwhile, interdisciplinary advances in multi-omics profiling, targeted genetic modification, and green extraction have enabled the development of multiple loquat tissues as raw materials for functional foods (Son et al., 2025; Moratilla-Rivera et al., 2026), botanical pharmaceuticals (Yazdanpanah et al., 2026), green biomaterials (Taktak and Gokce, 2026), and ecological fertilizers, demonstrating extraordinary diversification potential.

In traditional production, fresh loquat fruit constitutes the main commodity. However, large quantities of agricultural by-products, including fruit peels, seeds, senescent fallen flowers, and pruned branches, are arbitrarily discarded during field cultivation, fruit harvesting, and postharvest processing. This indiscriminate abandonment not only causes substantial loss of endogenous bioactive resources but also exacerbates environmental pollution derived from agricultural solid waste accumulation.

However, existing scholarly reviews and empirical investigations predominantly focus on individual bioactive compounds or single tissue types, while comparative profiling of tissue-specific metabolic signatures and functional partitioning across multiple loquat organs remains understudied. Current research paradigms often artificially separate the medicinal value of loquat leaves from the edible value of fruits, failing to elucidate the intrinsic chemical divergence and functional specificity among heterogeneous loquat organs. Such fragmented research frameworks obscure the molecular and material basis of loquat’s pharmacological potency and impede the rational allocation of bioactive resources. Accordingly, this review comprehensively collates the existing research findings and resource-utilization status of the five major loquat tissues—pulp, peel, seed, leaf, and flower—and clarifies the conserved features and tissue-specific distribution patterns of their bioactive metabolites. Meanwhile, targeting the practical industrial dilemmas, it dissects the realistic bottlenecks, including the fragmented processing layouts, the low-efficiency recycling of agricultural residual biomass, and the frost-induced instability of raw material supply, and accordingly proposes corresponding resource-transformation strategies, aiming to provide insights and references for the high-value exploitation and sustainable development of loquat germplasm and metabolically active resources.

## 2 Methods

Literature was retrieved from PubMed (n = 447) and the Web of Science Core Collection (n = 959) in June 2026 (no publication-date restrictions) using the unified core search terms “loquat” OR “*Eriobotrya japonica*”. The records retrieved from both databases were integrated into a single pool and deduplicated through a three-tier matching strategy (DOI, PubMed identifier, and normalized titles), and the literature inclusion and exclusion criteria were defined as a four-stage screening pipeline: (i) removal of duplicates (n = 257); (ii) removal of non-research document types, including meeting abstracts, conference proceedings, editorials, news, corrections, letters, retraction notices, and dissertations (n = 207); (iii) removal of records without abstracts (n = 28); and (iv) removal of non-English publications (n = 18). The search targeted loquat; findings from closely related wild species (e.g., *Eriobotrya deflexa* and *Eriobotrya* cavaleriei) are cited in the text where they inform tissue-specific metabolic comparisons. As a narrative/critical review, the retained records were synthesized by tissue type (pulp, peel, seed, leaf, and flower) and compound class, with priority given to peer-reviewed original studies and authoritative reviews, and the quality and limitations of the supporting evidence were critically appraised throughout (see Evidence grading below).

To map the research landscape, a bibliometric overview of the 763 retained English, peer-reviewed records was performed (1,406 records retrieved; 257 duplicates removed; 1,149 records screened; 253 excluded by the automated criteria above; 133 further excluded by thematic and bioinformatics screening; **Figure 1**). This quantitative analysis was conducted specifically to reveal structural gaps in tissue-specific loquat research. Among the 763 records, 50.3% focused on a single tissue (leaf 29.2%, seed 8.3%, flower 7.1%, pulp 3.1%, peel 2.6%), 10.7% (82 records) compared two or more tissues, and 38.9% were not tissue-specified. Among the 82 multi-tissue records, 81 (98.8%) were original research articles and one was a review (Kumar et al., 2024). Notably, single-tissue studies on peel and seed, which are largely treated as processing by-products, together account for less than 6% of the literature, and multi-tissue comparative studies are relatively uncommon, pointing to research gaps in the exploitation of these tissues and in integrated tissue-utilization strategies (**Figure 1**, right panel). All percentages were calculated with n = 763 as the denominator; each single-tissue record was counted once under its primary tissue, whereas multi-tissue records were classified exclusively into the multi-tissue category.

**Figure 1:**
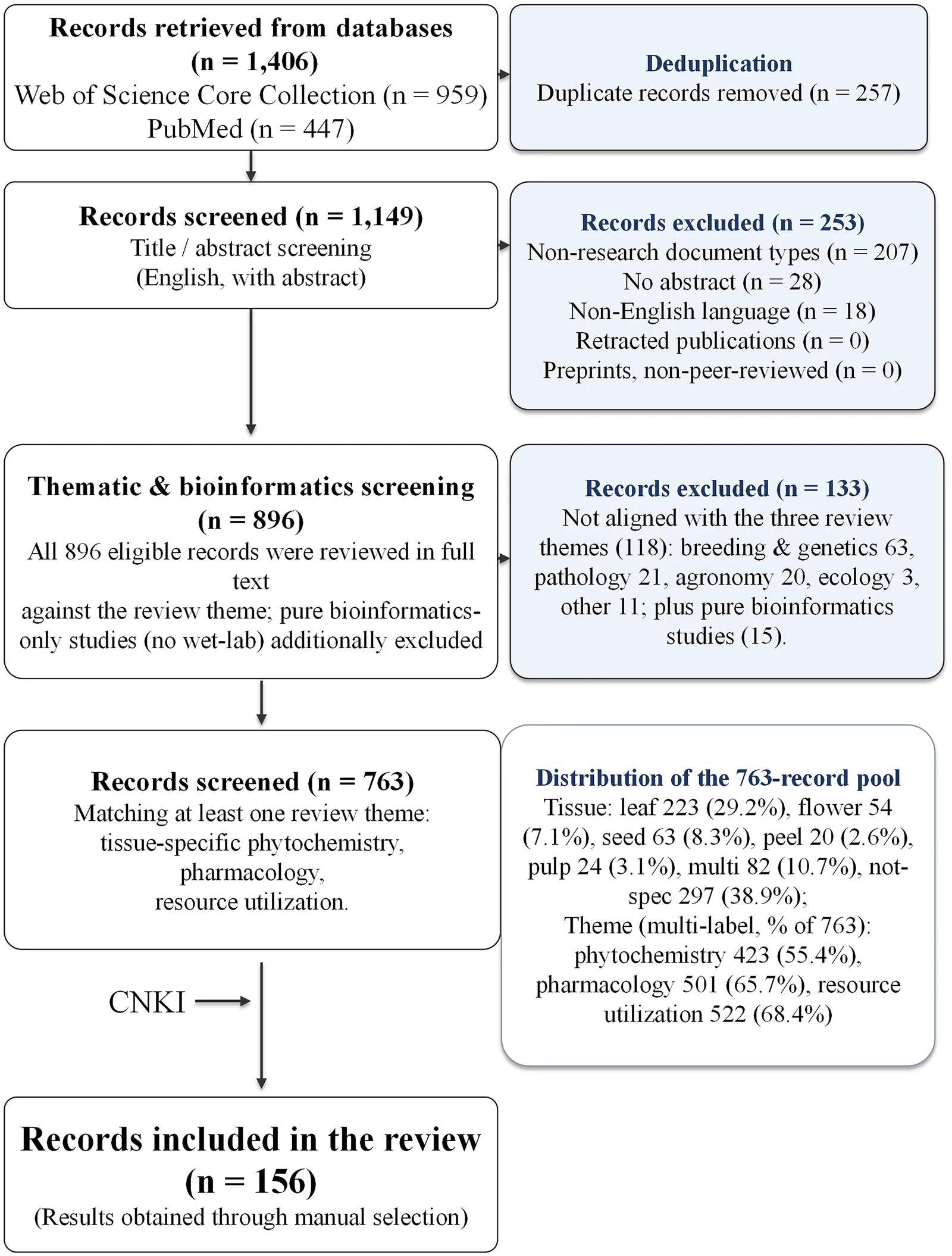
Flow diagram of the literature search and screening process. All records were retrieved from two academic databases: PubMed (n = 447) and Web of Science Core Collection (n = 959), with the unified core search terms “loquat” OR “*Eriobotrya japonica*”. All retrieved records were merged into a single dataset and underwent deduplication processing via a three-tier matching strategy (based on DOI, PubMed identifier and normalized titles. Subsequently, literature screening was conducted in five sequential stages: (1) Removal of duplicate records (n = 257); (2) Exclusion of non-research document types, records without abstracts, and non-English publications (n = 253); (3) Preliminary eligibility screening that retained English-language peer-reviewed records with complete abstracts (n = 896); (4) Three themes of “tissue-specific phytochemistry”, “pharmacology”, “resource utilization” screening was carried out centered on the review (with 133 papers excluded), ultimately yielding 763 records applicable for research direction trend analysis; (5) Following a manual review process and augmented by a search of the Chinese literature, 156 articles were identified and included in the present review.

The final reference set of this review was defined by two complementary routes. First, the 763 retained records were further prioritized by manual relevance screening and ranking against the review themes (tissue-specific phytochemistry, pharmacological functions, and resource-conversion mechanisms) and journal influence. Second, this English-language evidence base was supplemented with high-quality literature from the China National Knowledge Infrastructure (CNKI), which provides essential regional, historical, traditional-medicine, and practical processing context. CNKI records were not included in the quantitative analyses or percentage calculations described above; they were cited additionally and served as important supporting evidence for the discussion of pharmacological activities, tissue-specific utilization, and related viewpoints throughout this review.

Evidence grading. To avoid overstating phytochemical and pharmacological evidence, an explicit evidence hierarchy is adopted throughout this review. For phytochemical identification, three levels are distinguished: (i) compounds isolated and fully structurally characterized (e.g., by NMR and HR-MS); (ii) compounds identified by co-elution with authentic standards (e.g., HPLC/LC-MS with reference compounds); and (iii) compounds only tentatively annotated by spectrometric methods without authentic standards. Likewise, for biological activity, chemical/enzymatic assays, cell-based *in vitro* studies, animal (*in vivo*) studies, and clinical evidence are clearly distinguished, and no cross-level equivalence is implied; where a claim rests on a lower evidence level, this is indicated explicitly in the text and in the table notes.

## 3 Tissue-specific metabolic characteristics and industrial utilization status of loquat

### 3.1 Loquat pulp: core edible tissue with diversified processing and functional development potential

As a representative food-medicine homologous fruit, loquat pulp accumulates abundant essential amino acids, carotenoids (Zhou et al., 2007a; Sagar et al., 2020), and multiple mineral elements including calcium, manganese, iron, and potassium (Pareek et al., 2014). Its superior flavor profile and nutritional value render loquat widely popular in the global fresh fruit market. Furthermore, loquat pulp accumulates substantial ascorbic acid, vitamin A, ursolic acid, tartaric acid, oleic acid, quercetin, and other bioactive phytochemicals (**Figure 2**). These endogenous metabolites collectively confer potent free radical-scavenging and antioxidant capacity (Xu et al., 2014), endowing loquat pulp with broad application prospects in the research and development of functional nutritional foods.

**Figure 2:**
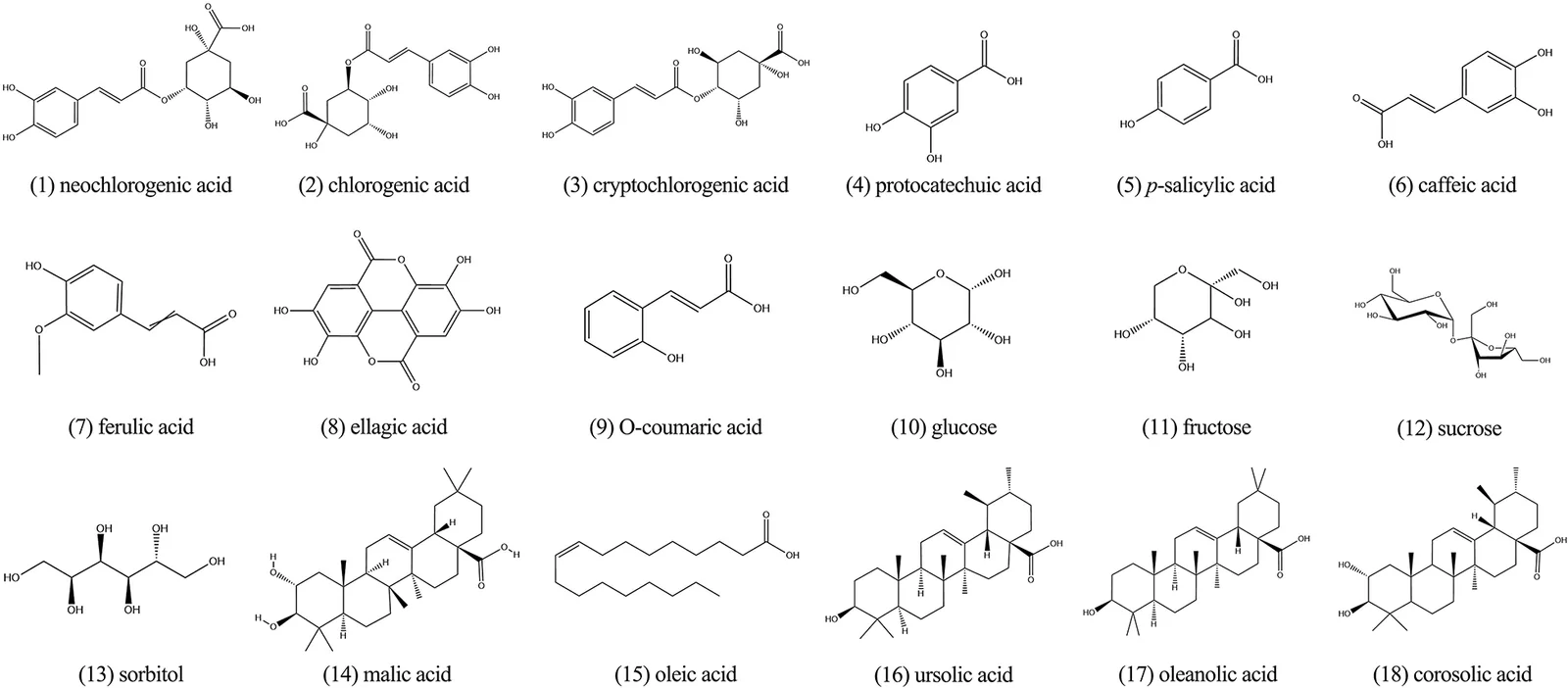
Some representative functional compounds in the flesh of loquat fruits. This image illustrates the chemical structures of 18 representative functional compounds identified in the flesh of loquat (*Eriobotrya japonica*) fruits, categorized by their chemical classes: Phenolic acids & derivatives (1-9): neochlorogenic acid, chlorogenic acid, cryptochlorogenic acid, protocatechuic acid, *p*-salicylic acid, caffeic acid, ferulic acid, ellagic acid, O-coumaric acid; Sugars & sugar alcohols (10-13): glucose, fructose, sucrose, sorbitol; Organic acid (14): malic acid; Fatty acid (15): oleic acid; Triterpenoids (16-18): ursolic acid, oleanolic acid, corosolic acid.

#### 3.1.1 Processing technology and edible products development

Loquat fruits ripen in early summer, characterized by low pulp firmness, 0.3%-0.6% titratable acidity, and soluble solid content exceeding 12% (Dhiman et al., 2021). Nevertheless, loquat exhibits very short shelf life under ambient temperature conditions and is highly susceptible to mechanical damage after harvesting. Postharvest mechanical injury easily induces water transpiration loss, nutrient degradation, and microbial rot, considerably restricting long-distance transportation and long-term commercial circulation (Shah et al., 2023). Current research on loquat fruit processing predominantly focuses on postharvest preservation technology optimization, fresh food processing, and intensive functional component extraction. Existing preservation studies mainly optimize the permeability of packaging materials, coating-preservative formulation, and postharvest pretreatment and storage environmental conditions (Cai et al., 2006; Cao et al., 2009). Chilling injury and pulp lignification are confirmed as the two key physiological bottlenecks limiting the postharvest storage and commercial circulation of loquat fruit (Zhang et al., 2022). Recent molecular physiological studies have gradually clarified the molecular regulatory mechanisms underlying loquat fruit lignification and cold tolerance. Low-temperature conditioning (LTC; e.g., conditioning at 5°C for 6 days before transfer to 0°C storage) can effectively suppress cold-induced lignification via modulating the *EjNAC3-EjNAC4* transcriptional cascade (Cai et al., 2006; Ge et al., 2017). In red-fleshed loquat, modified low-temperature conditioning (mLTC; 5°C for 2 days before 0°C storage (Cao et al., 2026), alleviates cold-triggered lignification through the *EjXND1*-mediated *EjHB1*-*EjPRX12* module, and the expression of *EjXND1* is dynamically regulated by DNA methylation levels in its promoter region (Liang et al., 2025). Exogenous supplementation of hydrogen sulfide, melatonin, and methyl jasmonate has also been validated to enhance pulp antioxidant enzyme activity and cold resistance, thereby delaying postharvest senescence and quality deterioration of loquat fruit (Shen et al., 2025).

Loquat pulp possesses excellent processing adaptability for diversified deep processing. Loquat juice, as a mainstream processed product, features low calorie density and rich accumulation of vitamins, polyphenols, flavonoids, and carotenoids, with verified potential for blood lipid regulation and cardiovascular protection (Meng et al., 2022). Optimization of juice production and clarification processes effectively extends the loquat industrial chain, and loquat juice can serve as a functional flavor modifier to enhance the sensory quality of beer products (Li et al., 2024). Endogenous soluble sugars in loquat pulp can also be utilized as natural carbon sources for fermenting characteristic fruit wine and fruit vinegar with unique aromatic profiles (Duan et al., 2018; Liu et al., 2024a).

Canned loquat represents another traditional and mainstream processed product that effectively preserves the morphological integrity of the fruit. However, dynamic variations in fruit maturity induce inconsistent accumulation of characteristic aroma components such as β-ionone, resulting in uneven flavor of canned products (Takahashi et al., 2000). The high endogenous pectin content of loquat pulp enables its application as a superior raw material for jam, jelly, preserved fruit, and functional fermented yogurt (Curi et al., 2016; Qiao et al., 2024). Integrated gas chromatography-ion mobility spectrometry (GC-IMS) and electronic sensory technology have been deployed to optimize loquat jam processing parameters, maximizing the retention of characteristic flavor substances (Qiao et al., 2024). Diversified intensive processing technologies convert perishable fresh loquat into storable commercial products, effectively extending product circulation cycles and significantly elevating the economic added value of loquat resources.

#### 3.1.2 Functional components and medicinal pharmacological attributes of loquat pulp

Loquat pulp synthesizes and accumulates a diverse spectrum of bioactive metabolites, mainly including phenolic derivatives, triterpenic acids, soluble sugars, and organic acids (Zhao, 2024). These phytochemicals constitute the fundamental substance basis for its dual dietary and therapeutic attributes. The phenolic profile of loquat pulp comprises neochlorogenic acid, chlorogenic acid, cryptochlorogenic acid, protocatechuic acid, *p-*salicylic acid, caffeic acid, ferulic acid, ellagic acid, o-coumaric acid and other phenolic derivatives (Ding et al., 2001). Chlorogenic acid dominates the phenolic composition of mature loquat fruit, while neochlorogenic acid and cryptochlorogenic acid act as core contributors to pulp antioxidant capacity (Xu et al., 2014).

Triterpenoids are crucial secondary metabolites in loquat pulp (Zhou et al., 2011b), predominantly existing as saponin derivatives synthesized from ursolic acid, oleanolic acid, and corosolic acid skeletons. Ursolic acid and oleanolic acid are structural isomers (Li et al., 2009) that naturally exist in free acid forms or serve as aglycones of triterpenoid saponins (Ayeleso et al., 2017), exhibiting prominent pharmacological activity and research translational value (Su et al., 2021). Soluble sugars and organic acids function as core quality-determining components that shape the overall flavor and commercial value of loquat fruit (Chen et al., 2009). Glucose, fructose, sucrose, and sorbitol constitute the major soluble sugars and sugar alcohols, while galactose is only detectable in immature fruits (Cao et al., 2013). During fruit ripening, fructose and sorbitol contents gradually decline, accompanied by continuous sucrose accumulation. Differences in sugar sweetness thresholds, dynamic metabolic variations across maturation stages, and the coexistence of organic acids jointly shape the unique flavor characteristics of fresh loquat and its processed derivatives.

#### 3.1.3 Functional transformation and application of pulp-derived bioactive substances

Modern separation and purification technologies, such as ultrasonic-assisted extraction, microwave extraction, and macroporous resin separation, enable high-efficiency enrichment of core functional components from loquat pulp (Liu et al., 2025b; Ozkan et al., 2024; Xiao et al., 2023). Triterpenic acids represented by corosolic acid exert definitive anti-inflammatory, antiviral, and hypoglycemic effects, which can be developed into specific efficacy health products (Xu et al., 2026). Compound functional foods can be fabricated by compounding loquat pulp extracts with prebiotics and vitamins to meet the nutritional intervention demands of the experimental subjects (Kumar et al., 2024). Loquat pulp polysaccharides possess prominent immunomodulatory and hypoglycemic bioactivities, they effectively activate macrophage immune responses and enhance human non-specific immune function. Purified loquat polysaccharides can be incorporated into conventional food matrices as functional fortifiers or further processed into oral liquids, capsules, and tablets for human immune regulation and sub-health state intervention (Sagar et al., 2020).

### 3.2 Loquat peel: a high-density repository of bioactive metabolites

As a frequently discarded by-product tissue, loquat peel accumulates a distinctive repertoire of phenolic, flavonoid, and triterpenoid metabolites (Zhang et al., 2015) that underpins both its pharmacological value and industrial potential. Loquat features a lengthy growth cycle and highly synchronized fruit ripening period, which restricts the sustainable annual supply of fresh commercial fruits. Although deep-processing products such as canned loquat effectively balance market supply–demand relationships and extend the industrial chain, these manufacturing procedures generate enormous peel by-products (Dhiman et al., 2021). Direct abandonment of loquat peel not only induces considerable agricultural bioresource waste but also exacerbates ecological pressure derived from solid waste pollution (Liu et al., 2025a). Accordingly, comprehensive elucidation of peel nutritional and functional components and promotion of high-value resource recycling are imperative for the green and sustainable development of the loquat processing industry.

Nutritional data from the USDA Nutrition Database (https://www.nutrition-and-you.com/loquat-fruit.html) demonstrate that loquat peel exhibits superior nutritional attributes compared with those of the pulp, with significantly higher accumulation of crude protein, vitamin C, carotenoids, and dietary fiber. Notably, the dietary fiber content of loquat peel reaches 47.2%, qualifying it as a high-purity natural dietary fiber resource. Loquat peel also contains abundant mineral elements and a well-balanced amino acid profile, which can be directly processed into dietary fiber fortifiers after low-temperature drying and ultrafine crushing for primary resource utilization.

#### 3.2.1 Characteristic bioactive components of loquat peel

Loquat peel develops from the ovary wall and provides effective protection for the fruit flesh tissue (Suhag et al., 2023). In addition to being rich in dietary fiber, it contains abundant high-functional compounds such as polyphenols, flavonoids, triterpenes, polysaccharides, and lipids (Liu et al., 2025a). Geographically representative loquat peel samples from Morocco exhibit high metabolic activity, with aqueous extracts containing 189.23 ± 0.84 mg/g total polyphenols and 103.45 ± 0.03 mg/g total flavonoids (Mokhtari et al., 2023b). The core phenolic constituents of loquat peel (**Figure 3**) include chlorogenic acid, neochlorogenic acid, 3-*p-*coumaroylquinone, 5-abeyloylquinone, and gallic acid. Except for chlorogenic acid, whose accumulation levels are consistent in the pulp and peel, the majority of phenolic compounds are significantly enriched in peel tissues (Ferreres et al., 2009; Suhag et al., 2023; Mokhtari et al., 2023b). The flavonoid profile of loquat peel comprises more than ten monomeric compounds (**Figure 3**), predominantly quercetin and kaempferol glycoside derivatives. Its total flavonoid content is markedly higher than that of pulp, second only to loquat leaves, establishing peel as a critical flavonoid-enriched tissue (Yan et al., 2023; Suhag et al., 2023; Mokhtari et al., 2023b).

**Figure 3:**
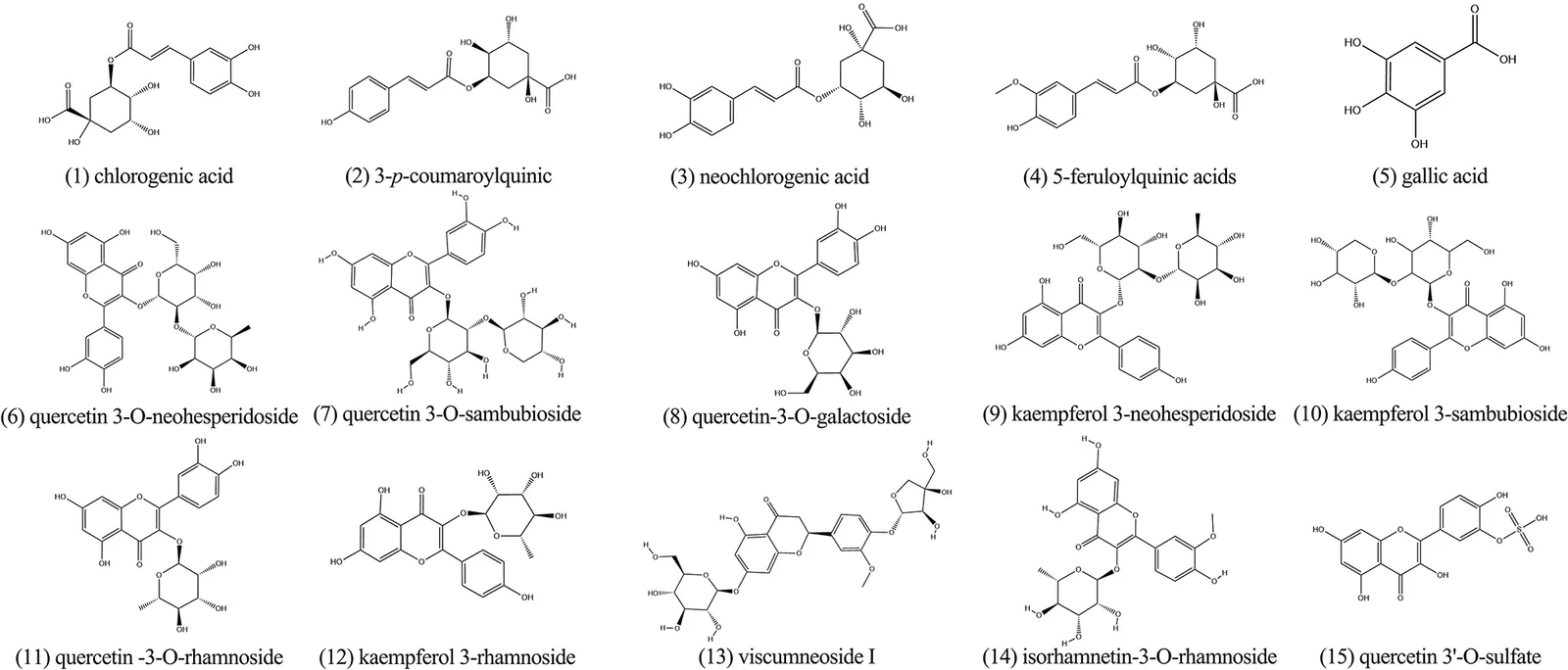
Some representative functional compounds in the peel of loquat fruits (Yan et al., 2023). This image displays the chemical structures of 15 representative functional compounds identified in the peel of loquat (*Eriobotrya japonica*) fruits, primarily belonging to the phenolic acid and flavonoid classes: Phenolic acid derivatives (1-5): chlorogenic acid, 3-*p-*coumaroylquinic acid, neochlorogenic acid, 5-feruloylquinic acid, gallic acid; Flavonoid glycosides (6–15): quercetin-3-O-neohesperidoside, quercetin-3-O-sambubioside, quercetin-3-O-galactoside, kaempferol-3-neohesperidoside, kaempferol-3-sambubioside, quercetin-3-O-rhamnoside, kaempferol-3-rhamnoside, viscumneoside I, isorhamnetin-3-O-rhamnoside, quercetin-3’-O-sulfate.

Triterpenoids constitute the primary pharmacologically active ingredients of loquat peel, with ursolic acid accounting for 51.4% of total peel triterpenic acids (Chen et al., 2022). Loquat peel polysaccharides (LPWP) possess high structural purity, stable methylated modification, and highly branched arabinopolysaccharide side chains, with *in vitro* functional assays confirming prominent antioxidant and metabolic regulatory activities (Liu et al., 2025a). Collectively, these enriched bioactive components endow loquat peel with considerable deep-processing and value-added transformation potential.

#### 3.2.2 Industrial transformation of peel-derived functional substances

The unique chemical composition and physicochemical properties of loquat peel enable its diversified application in functional food, natural beverage, and pharmaceutical industries (Costa et al., 2022). Peel aqueous extracts integrate polyphenolic, flavonoid, and triterpenoid components with a distinctive sweet-sour fruit aroma, serving as superior raw materials for natural functional beverage fabrication. Peel polysaccharides exhibit excellent *in vitro* antioxidant and hypoglycemic bioactivities, and can function as food emulsifiers, stabilizers, and prebiotics to optimize food system stability and nutritional functionality, supporting the development of targeted dietary supplements for metabolic disorder intervention.

Highly accumulated polyphenols, flavonoids, and ursolic acid in loquat peel exert potent antioxidant, lipid-regulating, cardiovascular-protective, and hypoglycemic effects. These bioactive compounds can be developed into botanical preparations for the auxiliary prevention and intervention of hyperlipidemia, hyperglycemia, and cardiovascular diseases. Full exploitation of loquat peel resources effectively resolves agricultural by-product waste and environmental pollution dilemmas, providing a feasible technical pathway for resource recycling and high-value industrial upgrading of loquat processing by-products.

### 3.3 Resource exploitation and functional industrialization of loquat seeds

Loquat seeds account for 20%-30% of the single fruit weight and constitute the predominant by-product of loquat deep processing (Al-Bachir and Koudsi, 2021; Amin et al., 2025). Seeds accumulate unique and specific bioactive components and exhibit substantial development potential in multiple domains such as medicine, food, and materials science, thereby manifesting notable potential for resource utilization (Chen et al., 2012).

#### 3.3.1 Chemical profiling and metabolic accumulation characteristics of loquat seeds

Loquat seeds harbor a rich and diverse spectrum of chemical constituents (**Figure 4**), with core bioactive substances encompassing cyanoglycosides (Sagar et al., 2020), polyphenols (Amin et al., 2025), triterpenic acids, and functional polysaccharides (Taskin et al., 2011; Zhou and Tan, 2025). These components exhibit stable accumulation that forms the material basis for multi-dimensional physiological functions (Kalderis et al., 2023). Cyanoglycosides are signature components of loquat seeds, with amygdalin as the dominant constituent localized in seed kernels. Amygdalin contains two stereoisomers, namely D-amygdalin and L-amygdalin (Xu et al., 2017). D-amygdalin exerts prominent anti-inflammatory and antitumor activities, while L-amygdalin possesses potential therapeutic value for cough and asthma intervention (Xu et al., 2017; He et al., 2020). The D-amygdalin content in loquat seeds is comparable to that of most Rosaceae species and significantly higher than non-Rosaceae plants, and its excellent thermal stability provides favorable conditions for industrial processing and utilization (Xu et al., 2017; Wu et al., 2017). Nevertheless, the safe utilization of loquat seeds must account for their cyanogenic glycoside content: upon enzymatic hydrolysis, amygdalin releases hydrogen cyanide (HCN), whose toxicity is strictly dose-dependent. The cyanogenic potential of loquat seeds, the effects of processing (e.g., heating, soaking, fermentation, and removal of the seed coat, which reduce β-glucosidase-mediated HCN release), and the available toxicological evidence therefore need to be evaluated before any food, feed, or nutraceutical application, and only processed products complying with established cyanide safety limits should be regarded as edible (He et al., 2020; Xu et al., 2017; He et al., 2020). Accordingly, the seed-derived applications discussed below should be read as development prospects pending rigorous safety validation rather than as established edible uses. The seed polyphenol pool further comprises caffeic acid, chlorogenic acid, gallic acid, catechins, epicatechins, and tannic acid (**Figure 4**), with caffeic and chlorogenic acids serving as core contributors to the antioxidant capacity (Pande and Akoh, 2010; He et al., 2020); the total phenolic content of dried seeds reaches 64.49 mg gallic acid equivalents/g, markedly exceeding that of peel and pulp (Amin et al., 2025). Immature seeds accumulate higher phenolic antioxidant levels, indicating greater resource-development potential (Al-Hassan and Norziah, 2012; Amin et al., 2025).

**Figure 4:**
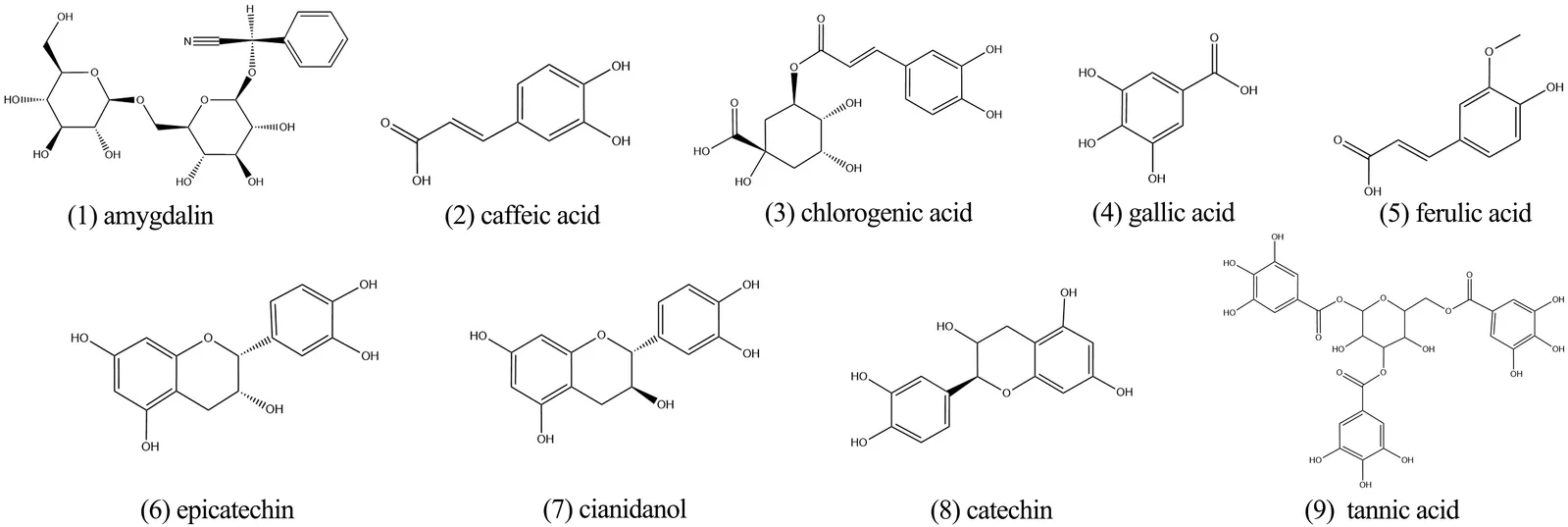
Some representative functional compounds in the seeds of loquat fruits. This image presents the chemical structures of 9 representative functional compounds identified in the seeds of loquat (*Eriobotrya japonica*) fruits, which can be categorized into distinct chemical classes: Cyanogenic glycoside: (1) amygdalin; Phenolic acids (2-5): caffeic acid, chlorogenic acid, gallic acid, ferulic acid; Flavan-3-ols (6-8): epicatechin, cianidanol (epicatechin enantiomer), catechin; Tannin (9): tannic acid — a hydrolyzable tannin with strong antioxidant and astringent properties.

The core triterpenic acid components of loquat seeds are ursolic acid and corosolic acid (Chen et al., 2022). Correlation analysis has revealed that the contents of arbutin and kaempferol both show highly significant positive correlations with total triterpenic acid content (correlation coefficients r = 0.87 and r = 0.78, respectively), and a significant positive correlation is also observed between the two components (r = 0.65), indicating stable accumulation patterns (Chen et al., 2022). Micellar capillary electrophoresis technology enables simultaneous quantitative detection of multiple seed active components, providing technical support for efficient component utilization (Weng and Zheng, 2010). Loquat seed polysaccharides prepared via conventional extraction methods exhibit excellent biosafety without significant cytotoxicity, laying a solid foundation for biomedical and functional food applications (Zhou and Tan, 2025).

#### 3.3.2 Multi-dimensional utilization of seed-derived bioactive substances

Benefiting from the rich chemical diversity, loquat seed metabolites exhibit extensive application prospects in medical health, functional food development, novel biomaterials, and biological fermentation, supporting multi-dimensional high-value transformation of agricultural by-product resources.

In pharmaceutical and healthcare domains, loquat seed bioactive components exert broad-spectrum pharmacological activities, including anti-inflammation (Shin et al., 2003), tumor intervention, antioxidation, cardiovascular protection, and metabolic modulation. Seed-derived cyanoglycosides alleviate macrophage-mediated inflammatory injury by suppressing NFκB and p38 MAPK signaling cascades and reducing pro-inflammatory cytokine transcription, with verified anti-inflammatory and immunomodulatory efficacy in asthma, radiation-induced inflammation, and rheumatoid arthritis models (Luo et al., 2020; Yang et al., 2013; Zhang et al., 2023). For tumor intervention, seed extracts inhibit AKT-mTOR pathway activation, block tumor cell cycle progression, and regulate apoptosis-related protein expression, thereby suppressing the proliferation and metastasis of bladder, renal, prostate, and cervical cancer cells (Makarević et al., 2014; Shi et al., 2019; He et al., 2020; Al-Khafajia and Tok, 2021). Loquat seed extracts exhibit remarkable free radical scavenging capacity against DPPH and reactive oxygen species in a concentration-dependent manner, conferring potent antioxidant activity (Pande and Akoh, 2010). The endogenous flavonoid constituents efficiently inhibit low-density lipoprotein oxidation. Combined with the inherent anti-atherosclerotic efficacy of amygdalin, these components produce synergistic cardiovascular protective effects, which effectively reduce the risk of cardiovascular diseases (Koba et al., 2007). In regard to metabolic modulation and organ protection, loquat seed extracts exert robust hypoglycemic effects, which have been validated in established type 2 diabetic rodent models (Tanaka et al., 2014).

In novel biomaterial research, loquat seed-derived non-traditional starch (LSS) possesses excellent biocompatibility and retains endogenous antioxidant phenolic components, enabling the fabrication of biodegradable films, functional surface coatings, and composite polymeric materials (Sharma et al., 2024). Seed extracts stabilize intracellular calcium homeostasis and delay fibroblast senescence, providing theoretical support for anti-aging skincare product development (Muramoto et al., 2010). In agricultural resource recycling, loquat seeds can serve as high-quality microbial culture substrates for scleroglucan production and α-amylase biosynthesis via solid-state fermentation, realizing harmless disposal and resource regeneration of processing by-products (Erdal and Taskin, 2010; Taskin et al., 2011).

### 3.4 Functional components and utilization value of loquat leaves

Among the five major loquat tissues, leaves accumulate the most diverse triterpenoid chemistry and possess prominent medicinal and health-care attributes, underpinning their pharmacological and industrial potential. Their rich reservoir of structurally diverse and highly active secondary metabolites mainly includes triterpenic acids, flavonoids, polyphenols, and polysaccharides.

#### 3.4.1 Characteristic functional ingredients of loquat leaves

Triterpenic acids constitute the predominant active components of loquat leaves, with senescent fallen leaves accumulating higher triterpenoid levels than mature leaves due to continuous metabolic accumulation during leaf development. Loquat leaves exhibit the most diverse triterpenoid composition and highest accumulation level among all loquat tissues, serving as optimal raw materials for triterpenic acid extraction (Chen et al., 2022). Multiple triterpenic acid compounds have been isolated and identified from loquat leaves (**Figure 5**), including oleanolic acid, maslinic acid, methyl arteminate, 2,3,23-trihydroxyolean-12-en-28-oic acid, δ-oleanolic acid, ursolic acid, methyl ursolate, corosolic acid, 3-O-cis-*p-*coumaroyltormentic acid, 3-O-trans-*p-*coumaroyltormentic acid, 3-epi-corosolic acid, euscaphic acid, 1β-hydroxyrosolic acid, ursolic acid lactone, betulinic acid and methyl betulinate (Banno et al., 2005).

**Figure 5:**
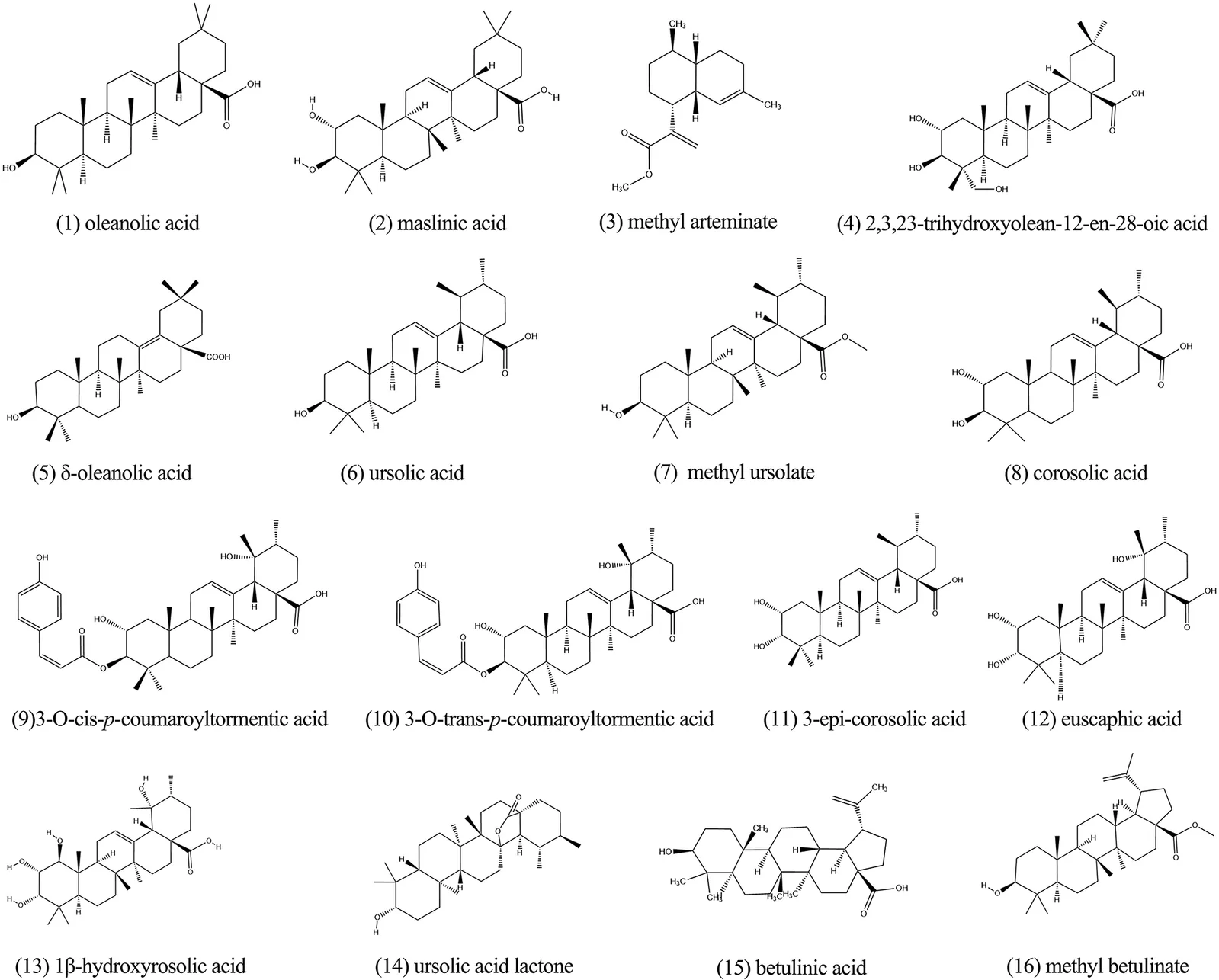
Some representative triterpenic acid compounds in loquat leaves (Banno et al., 2005). (1) oleanolic acid; (2) maslinic acid; (3) methyl arteminate; (4) 2,3,23-trihydroxyolean-12-en-28-oic acid; (5) δ-oleanolic acid; (6) ursolic acid; (7) methyl ursolate; (8) corosolic acid; (9) 3-O-cis-*p*-coumaroyltormentic acid; (10) 3-O-trans-*p*-coumaroyltormentic acid; (11) 3-epi-corosolic acid; (12) euscaphic acid; (13) 1β-hydroxyrosolic acid; (14) ursolic acid lactone; (15) betulinic acid (16) methyl betulinate.

The flavonoid composition of loquat leaves is dominated by kaempferol and quercetin aglycones, with glycoside derivatives modified by glucose, rhamnose, galactose, and arabinose residues (Xiao et al., 2023). Multiple flavonoid monomers and coumaroyl-modified flavonoid glycosides have been identified (Ito et al., 2002; Kim et al., 2025), endowing leaves with rich flavonoid metabolic diversity (**Figure 6**).

**Figure 6:**
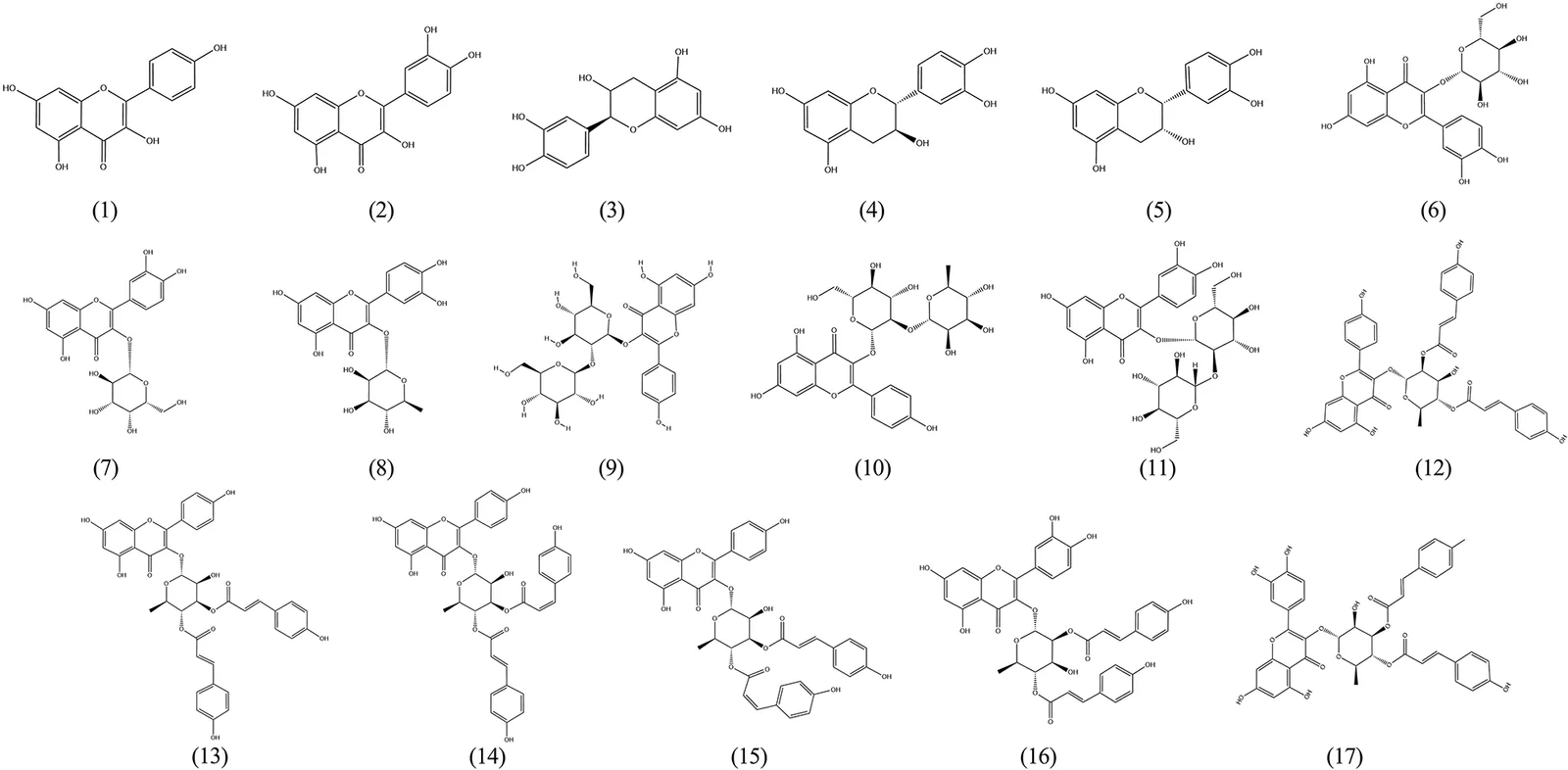
Some representative flavonoid compounds in loquat leaves (Kim et al., 2025). (1) kaempferol; (2) quercetin; (3) catechin; (4) epicatechin; (5) cinchonain; (6) quercetin-3-O-β-glucoside; (7) quercetin-3-O-β-galactoside; (8) quercetin-3-O-α-rhamnoside; (9) kaempferol-3-O-β-sophoroside; (10) kaempferol-3-neohesperidoside; (11) quercetin-3-O-sophoroside; (12) kaempferol-3-O-α-rhamnopyranoside 2”,4”-di-*E*-*p*-coumaroic acid ester; (13) kaempferol-3-O-α-(3”E,4”E di-*p*-coumaroyl)-rhamnoside; (14) kaempferol-3-O-α-rhamnoside 3”-*Z*,4”-*E*-di-*p*-coumaroic acid ester; (15) kaempferol-3-O-α-rhamnoside 3”-E,4”-*Z*-di-coumaroic acid ester; (16) quercetin 3-O-(2”,4”di-*E*-*p*-coumaroyl)-α-rhamnoside; (17) quercetin 3-O-(3”,”di-*E*-*p*-coumaroyl) -α-rhamnoside.

The polyphenolic profile of loquat leaves includes epicatechin, chlorogenic acid, procyanidin, cinchonain, and multiple coumaroylquinic acid derivatives (**Figure 7**), which collectively confer excellent antioxidant, anti-inflammatory, and antibacterial properties (Ito et al., 2000; Stefanos et al., 2022; Xiao et al., 2023). Beyond polyphenols, extraction methodologies significantly modulate the structural characteristics and bioactivities of loquat leaf polysaccharides (LLP). Polysaccharides obtained via hot water extraction and pressurized water extraction possess higher molecular weight and degree of esterification, while microwave-assisted and ultrasound-assisted extraction-derived polysaccharides exhibit superior antioxidant capacity and stronger inhibitory effects on carbohydrate digestive enzymes, which is closely associated with their enriched phenolic and flavonoid contents and unique molecular weight distribution (Fu et al., 2020).

**Figure 7:**
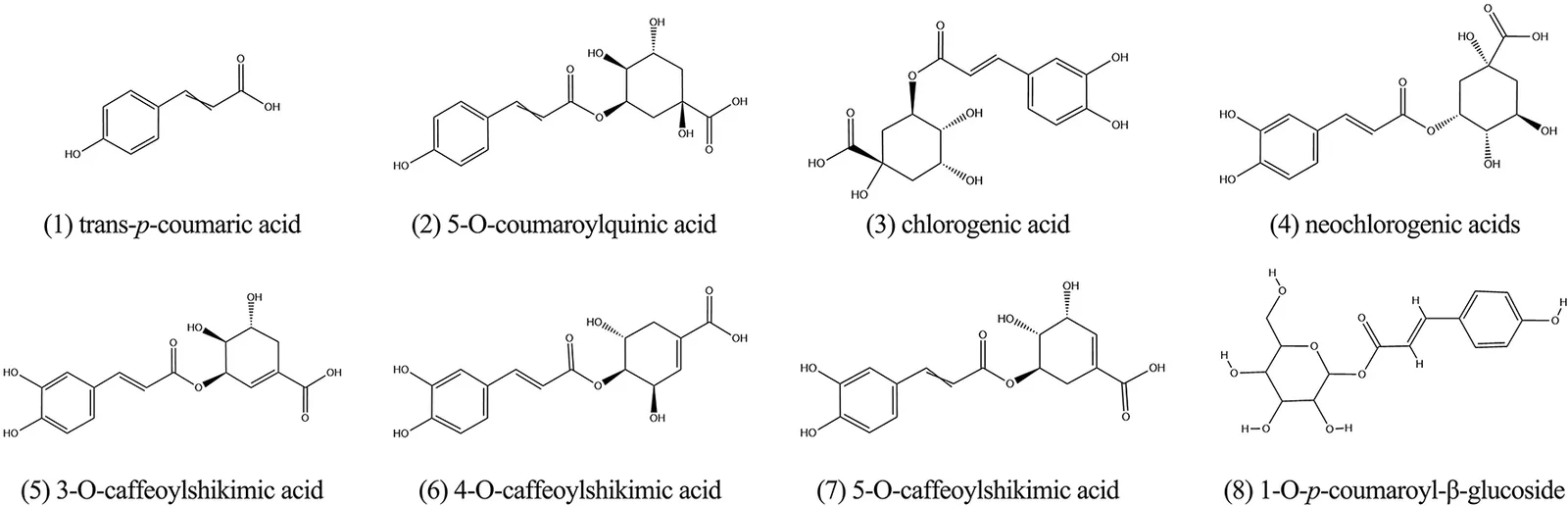
Some phenolic acid compounds in loquat leaves (Kim et al., 2025). (1) trans-*p*-coumaric acid; (2) 5-O-coumaroylquinic acid; (3) chlorogenic acid; (4) neochlorogenic acids; (5) 3-O-caffeoylshikimic acid; (6) 4-O-caffeoylshikimic acid; (7) 5-O-caffeoylshikimic acid; (8) 1-O-*p*-coumaroyl-β-glucoside.

#### 3.4.2 Pharmacological bioactivities of leaf-derived active components

Triterpenoid acids in loquat leaves are key antitumor substances (Ibrahim, 2021; Banno et al., 2005), which can effectively inhibit the growth of human gastric cancer BGC823 cells, and produce synergistic antitumor effects with maslinic acid and other components (Toshima et al., 2010; Lin et al., 2014). They suppress the invasion and metastasis of lung cancer and other tumor cells by inhibiting the activity and expression of matrix metalloproteinases MMP-2 and MMP-9 (Cha et al., 2011). Triterpenoid and polyphenolic components also inhibit the kinase activity of epidermal growth factor receptor (EGFR), providing novel candidate compounds for targeted tumor therapy (Ren et al., 2013).

In terms of glycemic regulation, leaf active ingredients inhibit α-amylase and α-glucosidase activity, modulate intestinal carbohydrate metabolism, and regulate intestinal flora structure to increase short-chain fatty acid production (Shih et al., 2010; Chen et al., 2021). Long-term intake of leaf extracts significantly ameliorates hyperglycemia, hyperinsulinemia and dyslipidemia in high-fat-diet mouse models. Corosolic acid exerts hypoglycemic effects through multiple mechanisms: improving insulin sensitivity, enhancing insulin signal transduction, regulating lipid metabolism, and reducing oxidative damage of pancreatic β-cells via antioxidant activity. Compared with clinical drugs gliclazide and metformin, loquat leaf triterpenoid acid extracts have little effect on fasting blood glucose of normal mice, but show stronger efficacy in inhibiting postprandial blood glucose elevation induced by oral glucose or starch (Li et al., 2011). Loquat leaf polysaccharides (LLP) bind to dietary fat, cholesterol, and bile acids *in vitro* and significantly inhibit carbohydrate digestive enzyme activity. *In vitro* digestion and fecal fermentation experiments confirm that leaf polysaccharides can be degraded and utilized by human intestinal microbiota, selectively enriching beneficial intestinal flora, including *Megasphaera, Megamonas, Bifidobacterium, Phascolarctobacterium, and Desulfovibrio* (Fu et al., 2019; Wu et al., 2021a). In addition, sesquiterpene glycosides isolated from loquat leaves possess remarkable hypoglycemic activity (Li et al., 2020a; Wu et al., 2022).

With respect to additional pharmacological properties, flavonoids extracted from loquat leaves ameliorate chemically-induced acute hepatic injury and inhibit the activation of hepatic stellate cells, thereby delaying the progression of hepatic fibrosis (Liang and Li, 2014). Leaf extracts also ameliorate liver and kidney dysfunction induced by viral infection and enhance immune antibody levels (He et al., 2013). Mixed triterpenic acids exert superior anti-inflammatory efficacy relative to single components by inhibiting the nuclear factor-κB (NFκB) signaling pathway and reducing nitric oxide (NO) production and the expression of inducible nitric oxide synthase (iNOS) and cyclooxygenase-2 (COX-2) (Cha et al., 2011). In terms of antibacterial properties, polyphenols in loquat leaves have strong inhibitory effects on bacteria and fungi. Their antibacterial activity against Candida albicans and Trichoderma is superior to potassium sorbate (Hong et al., 2011). Silver nanoparticles synthesized with loquat leaf extracts show good antibacterial activity against waterborne pathogens such as Listeria monocytogenes and Shigella (Hong et al., 2011).

#### 3.4.3 Diversified utilization of loquat leaves

The diverse bioactive components of loquat leaves support their dual development in edible functional products and medicinal preparations (Wu et al., 2003). Leaf-derived polyphenols, flavonoids, and sesquiterpene glycosides enable the development of fermented beverages, health teas, and functional chewing gum for chronic disease prevention and physical regulation (Toshima et al., 2010; Gao et al., 2021). In the field of traditional Chinese medicine, loquat leaf flavonoids, total triterpenic acids, and amygdalin serve as core ingredients in cough-suppressing and expectorant formulations. Classic Chinese patent medicines such as Loquat Paste, Loquat Cough Granules, and Compound Loquat Syrup have been widely employed in the clinical treatment of acute and chronic bronchitis, cough, and related conditions, with established market applications (Xin et al., 2006; Xiao et al., 2023). Additionally, the active components of loquat leaves exhibit significant antiemetic effects by reducing serotonin and dopamine levels in brain tissue, thereby alleviating cisplatin-induced vomiting (Zi and Zhang, 2016). In the field of modern drug development, the antitumor, metabolic regulatory, and organ-protective properties of active components in loquat leaves are continuously being explored. Ursolic acid C-3 amino derivatives can effectively inhibit the proliferation of leukemia cells, serving as promising candidates for targeted leukemia therapy (Ibrahim, 2021). Sesquiterpene glycosides are capable of modulating glucose and lipid metabolic pathways, and their extracts exert no adverse effects on normal blood glucose levels, thus emerging as excellent raw materials for the development of weight-loss products and novel antidiabetic agents (Li et al., 2020b). Moreover, the constituents of loquat leaves exhibit significant protective effects against conditions including nephritis, renal failure, gastric ulcers, and liver cirrhosis, suggesting substantial potential for the development of multi-organ protective drugs (Lu et al., 2013a; Zhu et al., 2022). In agricultural and cosmetic fields, loquat leaf extracts inhibit plant pathogenic bacteria and fungal spore germination for postharvest preservation, while amygdalin derivatives ameliorate skin inflammation and pigmentation, serving as natural functional cosmetic raw materials (Wang et al., 2012; Silva et al., 2020).

### 3.5 Active metabolites and developmental utilization of loquat flowers

Loquat flowers accumulate abundant kaempferol, ursolic acid, and oleanolic acid, with volatile metabolite profiles varying significantly across flowering developmental stages (Zhou et al., 2007b; Jiang et al., 2016). Volatile constituents are closely associated with aromatic characteristics, and their types and concentrations vary across different flowering stages. Using SPME-GC-MS technology, 49 aromatic compounds have been identified in loquat flowers, accounting for 93.34% of the total volatile substances (Zhang et al., 2008; Lu et al., 2013b; Shen et al., 2023). Loquat flowers are traditionally used to refresh the mind, moisten the lungs, relieve cough, eliminate phlegm, and soothe nerves, laying a foundation for their comprehensive development. Modern studies have shown that the water extract of loquat flowers exerts significant anti-inflammatory effects by inhibiting the production of prostaglandin E2 (PGE2) (Chen et al., 2023). Drinking loquat flower tea daily can clear away heat and detoxify, relieve respiratory discomfort, and moisten the throat. Processed loquat flower tea has reached a certain industrial scale in Zhejiang, Fujian, Sichuan and other regions of China (Ying et al., 2013). In traditional applications, loquat flowers are steamed with honey to treat wind-cold and cough, with the effects of dispelling wind, relieving cold and moistening throat. Loquat honey brewed from loquat flower nectar has an elegant fragrance and sweet taste, with the functions of moistening the lung, relieving cough and relaxing bowels. Flower volatile components can be developed as natural flavor additives for functional foods and daily chemical products (Shen et al., 2023), while flower polysaccharides with excellent moisturizing properties serve as natural skincare raw materials. Ecologically, loquat flower pollen and nectar attract pollinating insects, and insect feeding on flower secondary metabolites reduces pathogen transmission, forming a benign ecological cycle and diversified derivative industrial system (Nyska et al., 2014; Richardson et al., 2015).

## 4 Discussion

The tissue-level surveys presented above confirm that the five major loquat tissues share a conserved core of triterpenoid acids, phenolic acids, and flavonoids, while each organ accumulates signature compounds with distinct pharmacological profiles. Building on these compositional and functional findings, this section first discusses the cross-tissue metabolite distribution and the evolutionary and regulatory basis of tissue-specific metabolism, and then proposes four transformation strategies—marker-assisted germplasm improvement, multi-omics dissection of tissue-specific regulatory networks, synthetic-biology-based green biosynthesis of high-value phytochemicals, and a comprehensive circular industrial system—to overcome the three pivotal industrial constraints identified above: fragmented processing layouts, low-efficiency recycling of agricultural residual biomass, and frost-induced instability of raw material supply. Beyond these industrial constraints, the bibliometric analysis in Methods also exposes two evidence-level research gaps—the near-absence of human clinical trials and the lack of commercially marketed loquat functional products—which are elaborated in the Limitations section below.

### 4.1 Composition analysis among tissues

Loquat exhibits distinct tissue-specific accumulation patterns of secondary metabolites across the pulp, peel, seed, leaf, and flower, while maintaining a conserved core set of chemical components among different organs (Wang, 2021). **Table 1** summarizes the common compounds present in different loquat tissues and their core biological activities.

**Table 1:** Common compounds in various tissues of loquat (active ingredients commonly found in all tissues).

Class	Compound (molecular formula)	Isolation identification method	Biological function	References
Triterpenoid acids	Ursolic acid (C_30_H_48_O_3_)	L1: isolated, NMR (leaf; Banno et al., 2005); L2: HPLC (multi-tissue quantification; Chen et al., 2022)	Anti-inflammatory (NF-κB/MAPK/PDE4D); Antitumor	(Banno et al., 2005); (Zhou et al., 2011b)
	Oleanolic acid (C_30_H_48_O_3_)	L1: isolated, NMR (leaf); L2: HPLC	Anti-inflammatory; Antitumor; Hepatoprotective	(Banno et al., 2005); (Chen et al., 2022)
	Corosolic acid (2α-hydroxyursolic acid) (C_30_H_48_O_4_)	L1: isolated, NMR (leaf/callus; Ho et al., 2010); L2: HPLC (Chen et al., 2022)	Anti-inflammatory; Hypoglycemic (α-Glucosidase inhibition); Antitumor	(Ho et al., 2010); (Chen et al., 2022); (Li et al., 2009)
Flavonoids	Quercetin (C_15_H_10_O_7_)	L1: isolated (leaf flavonoids, hydrolysis; Ito et al., 2002); L2: HPLC	Antioxidant; anti-inflammatory; immunomodulatory; hypoglycemic	(Ito et al., 2002); (Zhou et al., 2011a)
	Kaempferol (C_15_H_10_O_6_)	L1: isolated (leaf/flower); L2: HPLC	Antioxidant; anti-inflammatory; immunomodulatory	(Ito et al., 2002); (Zhou et al., 2011a)
Phenolic acids	Chlorogenic acid (5-caffeoylquinic acid) (C_16_H_18_O_9_)	L2: HPLC with authentic standards (pulp/peel/seed/leaf)	Antioxidant; anti-inflammatory (NF-κB/p38 MAPK); hypoglycemic	(Ding et al., 2001); (Ge et al., 2009); (Cha et al., 2011)
	Neochlorogenic acid (C_16_H_18_O_9_)	L2: HPLC with authentic standards	Antioxidant (dominant phenolic in young fruit)	(Ding et al., 2001)
	Caffeic acid (C_9_H_8_O_4_)	L2: HPLC with authentic standards	Antioxidant; anti-inflammatory; antimicrobial	(Ding et al., 2001); (Song et al., 2010)
	Gallic acid (C_7_H_6_O_5_)	L2: HPLC with authentic standards	Antioxidant; anti-inflammatory	(Ferreres et al., 2009); (Ge et al., 2009); (Liu et al., 2025b)
Sugars	Glucose (C_6_H_12_O_6_)	L2: HPLC-RI/enzymatic quantification	Flavor (sweetness); energy metabolism	(Cao et al., 2013); (Liu et al., 2024a)
	Fructose (C_6_H_12_O_6_)	L2: HPLC-RI	Flavor (high sweetness)	(Cao et al., 2013); (Liu et al., 2024a)
	Sucrose (C_12_H_22_O_11_)	L2: HPLC-RI	Flavor (accumulated during ripening)	(Cao et al., 2013); (Liu et al., 2024a)
Sugar alcohols	Sorbitol (C_6_H_14_O_6_)	L2: HPLC-RI	Flavor; osmotic regulation	(Cao et al., 2013)
Organic acids	Malic acid (C_4_H_6_O_5_)	L2: HPLC with authentic standards	Flavor (acidity); processing adaptability	(Liu et al., 2024b); (Liu et al., 2024a)

Phytochemical identification evidence for the listed compounds is graded as (i) isolated and fully structurally characterized, (ii) identified with authentic standards, or (iii) tentatively annotated by spectrometric methods (see Methods, Evidence grading); biological activities are reported at chemical/enzymatic, cellular, or animal level as indicated by the cited references, and no clinical evidence is available for any compound.

Comparative analysis across the five major loquat tissues confirms that triterpenoid acids (ursolic acid, oleanolic acid and corosolic acid), phenolic acids (chlorogenic acid, neochlorogenic acid, caffeic acid and gallic acid) and flavonoids (quercetin and kaempferol) are the core active substances across all organs. Their universal distribution underpins the integral medicinal efficacy and edible health value of loquat, including anti-inflammatory effects, antioxidant activity, organ protection, and blood glucose reduction. By contrast, carbohydrates and organic acids, as fundamental primary metabolites in all loquat tissues, are not characteristic active ingredients, but critically determine the flavor and processing adaptability of each tissue (Liu et al., 2024b).

Notably, beyond these shared metabolites, loquat secondary metabolites exhibit prominent tissue specificity. Certain compounds are exclusively distributed in a single tissue or accumulate at significantly higher levels in specific organs. These tissue-unique components are closely associated with the specific ecological functions and medicinal characteristics of the corresponding loquat organs (**Tables 2**, **3**). For example, loquat peel polysaccharide (LPWP), a methyl-esterified pectin polysaccharide rich in branched arabinans, exhibits notable antioxidant and hypoglycemic activities *in vitro*, particularly demonstrating significant inhibitory effects against α-amylase and α-glucosidase (Liu et al., 2025a). Moreover, multiple bioactive components coexist in loquat tissue extracts, endowing the extracts with a variety of pharmacological activities. These bioactive molecules enter cells through receptor-mediated transport or passive diffusion, and participate in the activation of core signaling pathways such as NFκB, Nrf2 and Akt; additionally, they can act directly as small-molecule inhibitors to participate in endogenous enzymatic reactions and modulate cellular metabolic processes, ultimately exerting a series of pharmacological effects including antioxidant, anti-inflammatory, anti-infective, antitumor, anti-diabetic activities, as well as cell repair and tissue protection (**Figure 8**).

**Table 2:** Representative bioactive compounds reported in loquat tissues (pulp, peel, seed).

Tissue	Class	Compound (molecular formula)	Isolation identification method	Biological function	References
Pulp	Phenolic acids	Chlorogenic acid (predominant in ripe pulp) (C_16_H_18_O_9_)	L2: HPLC with authentic standards (content rises markedly at ripening)	Antioxidant	(Ding et al., 2001)
		Cryptochlorogenic acid (4-caffeoylquinic acid) (C_16_H_18_O_9_)	L2: HPLC with authentic standards	Antioxidant	(Ding et al., 2001)
		Ellagic acid (C_14_H_6_O_8_)	L2: HPLC (pulp phenolic profiling)	Antioxidant	(Xu et al., 2014)
		5-Feruloylquinic acid (C_17_H_20_O_9_)	L2: HPLC with authentic standards	Antioxidant	(Ding et al., 2001)
		4-*p*-Coumaroylquinic acid (C_16_H_18_O_8_) (flesh-specific; detected only in pulp)	L3: LC-MS profiling	Antioxidant	(Yan et al., 2023)
	Flavonoids	Chrysosplenoside B (flavone glycoside) (flesh-specific; detected only in pulp)	L3: LC-MS annotation	Antioxidant	(Yan et al., 2023)
	Aromatic volatiles	β-Ionone (C_13_H_20_O)	L3: GC-MS library matching	Characteristic aroma of pulp/canned loquat	(Takahashi et al., 2000)
Peel	Phenolic acids	Neochlorogenic acid (predominant in ripe pulp) (C_16_H_18_O_9_)	L2: HPLC with authentic standards	Antioxidant; anti-inflammatory	(Zhang et al., 2015)
		3-*p-*Coumaroylquinic acid (C_16_H_18_O_8_)	L2: HPLC (standards/spectral comparison)	Antioxidant	(Ferreres et al., 2009)
		5-O-Feruloylquinic acid (C_17_H_20_O_9_)	L2: HPLC	Antioxidant	(Ferreres et al., 2009)
		3-Caffeoylquinic acid (C_16_H_18_O_9_)	L2: HPLC	Antioxidant	(Ferreres et al., 2009)
	Flavonoid glycosides	Quercetin-3-O-neohesperidoside (C_27_H_30_O_16_)	L3: LC-MS annotation	Antioxidant	(Ferreres et al., 2009)
		Quercetin-3-O-sambubioside (C_26_H_28_O_16_)	L3: LC-MS annotation	Antioxidant	(Ferreres et al., 2009)
		Hyperoside (quercetin-3-O-galactoside) (C_21_H_20_O_12_)	L3: LC-MS annotation	Antioxidant; anti-inflammatory	(Ferreres et al., 2009)
		Quercitrin (quercetin-3-O-rhamnoside) (C_21_H_20_O_11_)	L3: LC-MS annotation	Antioxidant	(Ferreres et al., 2009)
		Isoquercitrin (quercetin-3-O-glucoside) (C_21_H_20_O_12_)	L3: LC-MS annotation	Antioxidant; anti-inflammatory	(Zhang et al., 2015)
		Kaempferol-3-O-neohesperidoside (C_27_H_30_O_15_)	L3: LC-MS annotation	Antioxidant	(Ferreres et al., 2009)
	Polysaccharides	LPWP (loquat peel pectic polysaccharide) (no fixed formula)	L2/L3: compositional and methylation analyses	Antioxidant; hypoglycemic (α-amylase/α-glucosidase inhibition)	(Liu et al., 2025a, b)
	Dietary fiber	Dietary fiber (cellulose; 47.2% of peel) (no fixed formula)	L2: compositional analysis (USDA FoodData Central)	High-purity natural dietary fiber resource	USDA FoodData Central (loquat)
	Tannins	Tannin (C_76_H_52_O_46_)	L2: chemical analysis	Antioxidant; astringency	(Mokhtari et al., 2023a)
Seed	Cyanogenic glycosides	D-Amygdalin (signature seed component) (C_20_H_27_NO_11_)	L1/L2: isolated and chromatographically quantified	Anti-inflammatory (NF-κB/p38); antitumor; dose-dependent HCN release on hydrolysis (safety)	(He et al., 2020); (Wang et al., 2020)
		L-Amygdalin (C_20_H_27_NO_11_)	L1/L2: isolated and chromatographically quantified	Potential antitussive value (traditional use; safety caveat as above)	(He et al., 2020)
	Polyphenols	Catechin (C_15_H_14_O_6_)	L2: HPLC with authentic standards	Antioxidant (contributes to high seed phenolic content)	(Amin et al., 2025); (Pande and Akoh, 2010)
		Epicatechin (C_15_H_14_O_6_)	L2: HPLC with authentic standards	Antioxidant	(Amin et al., 2025); (Pande and Akoh, 2010)
		Gallic acid (C_7_H_6_O_5_)	L2: HPLC with authentic standards	Antioxidant	(Amin et al., 2025)
		Protocatechuic acid (C_7_H_6_O_4_)	L2: HPLC with authentic standards	Antioxidant	(Amin et al., 2025)
	Tannins	Tannic acid (C_76_H_52_O_46_)	L2: chemical analysis	Antioxidant; astringency	(Amin et al., 2025)
	Starch	LSS (lignified starch from seeds) (no fixed formula)	L2: physicochemical characterization	Biodegradable films/composite materials	(Sharma et al., 2024)

Phytochemical identification evidence for the listed compounds is graded as (i) isolated and fully structurally characterized, (ii) identified with authentic standards, or (iii) tentatively annotated by spectrometric methods (see Methods, Evidence grading); biological activities are reported at chemical/enzymatic, cellular, or animal level as indicated by the cited references, and no clinical evidence is available for any compound.

**Table 3:** Representative bioactive compounds reported in loquat tissues (leaf and flower).

Tissue	Class	Compound (molecular formula)	Isolation/identification method	Biological function	References
Leaf	Triterpenoid acids	Maslinic acid (high abundance in leaf) (C_30_H_48_O_4_)	L1: isolated, NMR (leaf/callus)	Anti-inflammatory (NF-κB/MAPK); antitumor; hypoglycemic	(Banno et al., 2005); (Ho et al., 2010)
		Tormentic acid (C_30_H_48_O_5_)	L1: isolated, NMR	Anti-inflammatory; antidiabetic	(Ho et al., 2010)
		Euscaphic acid (C_30_H_48_O_5_)	L1: isolated, NMR	Anti-inflammatory; antitumor-promoting (*in vivo* two-stage carcinogenesis inhibition)	(Banno et al., 2005)
		Betulinic acid (C_30_H_48_O_3_)	L1: isolated, NMR	Anti-inflammatory; antitumor	(Banno et al., 2005)
		Ursolic acid lactone (C_30_H_48_O_3_)	L1: isolated, NMR	Anti-inflammatory	(Banno et al., 2005)
		3-O-cis/trans-*p-*Coumaroyltormentic acid (C_39_H_54_O_7_)	L1: isolated, NMR (leaf/callus)	Anti-inflammatory (*p-*coumaroylation increases lipophilicity)	(Banno et al., 2005); (Ho et al., 2010)
	Polyphenols	Procyanidin B-2 (dimer) (C_30_H_26_O_12_)	L1: isolated, NMR (leaf)	Antioxidant; antitumor (EBV-EA inhibition)	(Ito et al., 2000); (Ito et al., 2002)
		Procyanidin oligomers ((C_30_H_26_O_12_)n)	L1/L3: isolated and MS-characterized	Antioxidant	(Ito et al., 2000)
		Cinchonain Ia (C_24_H_20_O_9_)	L1: isolated, NMR (leaf)	Antioxidant; anti-inflammatory	(Ito et al., 2000)
		Cinchonain IIb (C_39_H_32_O_15_)	L1: isolated, NMR (leaf)	Antioxidant	(Ito et al., 2000)
	Flavonoids	Coumaroylated flavonol monorhamnosides (AFMR; di-*p-*coumaroyl esters of kaempferol/quercetin-3-O-rhamnoside) (C_39_H_32_O_15_ etc.)	L1: isolated (Ito et al., 2002); L3: LC-MS annotation (Kim et al., 2025)	Antioxidant; anti-inflammatory (high structural diversity)	(Ito et al., 2002); (Kim et al., 2025)
	Terpene glycosides	Sesquiterpene glycosides (leaf signature) (no fixed formula)	L1: isolated (leaf)	Hypoglycemic; metabolic regulation (α-glucosidase inhibition)	(Li et al., 2020a); (Gai et al., 2022)
Flower	Phenolic acids	*p-*Hydroxybenzoic acid (C_7_H_6_O_3_)	L2: HPLC with authentic standards	Antioxidant; preservative (cosmetic/pharmaceutical use)	(Zhou et al., 2011a)
		Salicylic acid (C_7_H_6_O_3_)	L2: HPLC with authentic standards	Anti-inflammatory; keratolytic	(Zhou et al., 2011a)
	Flavonoids	Kaempferol (high abundance in flower) (C_15_H_10_O_6_)	L2: HPLC with authentic standards	Antioxidant; anti-inflammatory	(Zhou et al., 2011a)
	Triterpenoid acids	Ursolic acid/Oleanolic acid (accumulated in flower) (C_30_H_48_O_3_)	L2: HPLC with authentic standards	Anti-inflammatory	(Zhou et al., 2007b)
	Volatile compounds	Floral volatiles (49 compounds: alcohols 13.2%, aldehydes 16.6%, esters 8.18%, etc.) (Mixture)	L3: SPME-GC-MS library matching	Natural flavor additives; fragrance	(Zhang et al., 2008); (Lu et al., 2013b); (Shen et al., 2023)
	Polysaccharides	Moisturizing polysaccharide (no fixed formula)	L2: compositional analysis	Moisturizing (cosmetic raw material)	(Chen et al., 2023); (Ying et al., 2013)

phytochemical identification evidence for the listed compounds is graded as (i) isolated and fully structurally characterized, (ii) identified with authentic standards, or (iii) tentatively annotated by spectrometric methods (see Methods, Evidence grading); biological activities are reported at chemical/enzymatic, cellular, or animal level as indicated by the cited references, and no clinical evidence is available for any compound.

**Figure 8:**
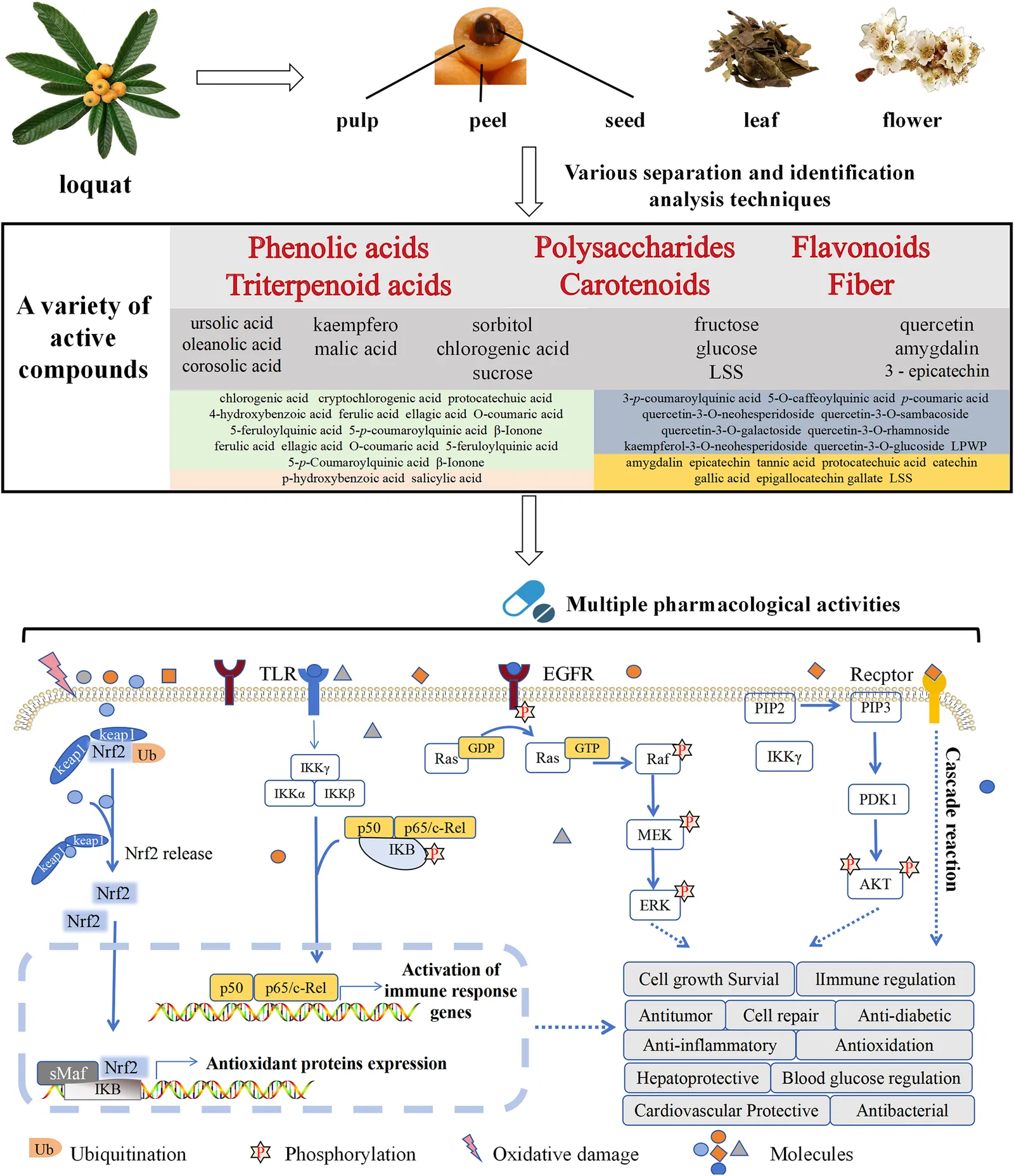
Multiple pharmacological activities of loquat tissues. TLR, Toll-like receptors; EGFR, epidermal growth factor receptor; Receptor, other receptors not marked. This schematic diagram summarizes the active compounds identified from different tissues of loquat (pulp, peel, seed, leaf, and flower) through various separation and identification techniques. The main classes of compounds include phenolic acids, triterpenoid acids, polysaccharides, carotenoids, and flavonoids. These bioactive constituents are associated with a wide range of pharmacological activities, such as antitumor, anti-inflammatory, antioxidant, immunoregulatory, hepatoprotective, and anti-diabetic effects. The lower panel illustrates the underlying molecular mechanisms, involving the activation of the Nrf2 pathway for antioxidant protein expression, the NF-κB pathway for immune response gene activation, as well as the EGFR/Ras/Raf/MEK/ERK and PI3K/AKT signaling cascades that regulate cell growth, survival, and other cellular functions. The results are derived from cell experiments or animal experiments and have not been verified by clear clinical data.

Evolutionary genomic analyses have demonstrated that the apple tribe has experienced a lineage-specific whole-genome duplication (WGD) event occurring approximately 13.5–27.1 Mya year. This genomic polyploidization event has been proposed to have driven the functional divergence and retention of gene clusters associated with triterpenoid biosynthesis, including key genes such as *EjOSC* and *EjCYP716A/C*. The duplicated paralogous copies of these genes constitute a critical molecular foundation for the markedly higher accumulation levels of total triterpenoids in mature loquat leaves relative to fruits, pulp, and seeds (Su et al., 2021). It should be noted that the WGD evidence derives primarily from genomic analyses of the apple tribe (Maleae), and direct experimental demonstration of a causal WGD–triterpenoid relationship in loquat itself remains to be established; the scenario presented here is therefore a working hypothesis. Further investigations into cold-responsive regulatory networks have uncovered a tissue-specific hierarchical cascade triggered by low-temperature signals. In leaf tissues, the transcription factor *EjCBF* positively regulates the expression of the downstream antioxidant enzyme-encoding gene *EjGST*, which consequently promotes the biosynthesis of antioxidant metabolites (Ma et al., 2024, 2025). During the early developmental stages of loquat fruits and seeds, the calcium signaling hub *EjCDPK25* remodels secondary metabolic pathways under freezing stress, driving the selective enrichment of seed-specific bioactive components (Xiong et al., 2024). Integrated hormonal and transcriptomic profiling has further validated that pivotal regulatory factors exemplified by EjHsc70 mediate hormone-dependent modulation during loquat fruit development. These factors facilitate pulp cell proliferation and the accumulation of soluble sugars and organic acids, while repressing the excessive biosynthesis and accumulation of triterpenoids and flavonoids. This dual regulatory mechanism partially elucidates the inherently low abundance of bioactive medicinal metabolites in the edible flesh of loquat fruits (Lin et al., 2025).

### 4.2 Exploiting wild germplasm resources to overcome bottlenecks in seed virus-free processing and high-value utilization

China, the center of origin and core distribution area of the loquat genus, harbors abundant wild germplasm with significantly higher contents of functional compounds than cultivated varieties. Research has demonstrated that wild loquat germplasm exhibits significantly elevated levels of triterpenoids, flavonoids, and cyanogenic glycosides relative to cultivated varieties, with arbutin content in wild loquat leaves and their hybrid progeny reaching 2–4 times that of cultivated varieties (Lin et al., 2022). However, these highly active wild loquat germplasm resources have not been comprehensively investigated or effectively harnessed. Consequently, future research on germplasm innovation and breeding in loquats should prioritize several key directions. At the level of germplasm identification and screening, established multi-parameter high-performance liquid chromatography techniques can be utilized to accurately identify distinctive loquat germplasms appropriate for medicinal development and functional applications. Concurrently, comprehensive plastid genome molecular marker toolkits for loquat can enable rapid and precise germplasm identification, traceability of regional specialty varieties, and high-throughput screening of terpene-derived active components in wild loquats (Lin et al., 2024, 2026). In variety improvement breeding, key cold-resistant functional genes identified through multi-tissue stress response assays (*EjCBF, EjCDPK25, EjGST*) can be employed in molecular-assisted breeding to develop frost-resistant, high-yielding, and stable-yielding loquat varieties, thereby mitigating yield and economic losses induced by winter frost disasters (Ma et al., 2024, 2025; Lin et al., 2026). Alternatively, through comparative genomic analysis, the elucidated *EjOSC-EjCYP716* metabolic pathway can act as a core breeding target for the genetic improvement of cultivated loquat. This pathway facilitates the breeding of loquat varieties with elevated triterpenoid accumulation, thereby improving the medicinal and commercial value of the species (Su et al., 2021).

### 4.3 Application of multi-omics technologies to elucidate tissue-specific metabolic differentiation mechanisms

Integrated multi-omics methodologies, encompassing genomics, transcriptomics, and metabolomics, have established a foundation for constructing tissue-component-function regulatory networks (Gómez-Espinoza et al., 2025). Subsequent research should integrate broad-spectrum targeted metabolomics with transcriptomic analyses to identify key rate-limiting enzymes and transcription factors involved in the biosynthesis of triterpenoids, flavonoids, and amygdalin in fruit peels and seeds. This would help elucidate the molecular mechanisms underlying the low content of bioactive compounds in edible flesh tissues and furnish molecular markers and breeding targets for targeted genetic improvement of by-products. Furthermore, metabolomics enables dynamic monitoring of the distribution dynamics of thermosensitive polyphenols and triterpenoids during extraction and fermentation processes, thereby aiding in the optimization of sustainable green processing technologies.

### 4.4 Establish a synthetic biology platform to achieve green biosynthesis of active monomers

High-value triterpenoids and flavonoids are primarily extracted from loquat tissues, which are constrained by seasonal raw material availability and high costs. Synthetic biology enables heterologous synthesis through the construction of microbial cell factories (yeast or Escherichia coli), eliminating reliance on agricultural cultivation (Cravens et al., 2019). Comprehensive identification of the triterpenic acid synthase gene family has been achieved by integrating transcriptomic analysis with co-expression profiling, yielding two oxidosqualene cyclase (OSC) genes and their associated modification genes. Through the application of a tobacco transient expression system, the existence of two independent ursane-type triterpenic acid biosynthesis pathways in loquat has been confirmed, thereby providing critical evidence for the development of natural small-molecule compounds via synthetic biology strategies (Liu et al., 2020; Su et al., 2021).

### 4.5 Establish a closed-loop, comprehensive circular industrial chain that covers all components

The global loquat industry has expanded rapidly in recent years, with the market size reaching USD 3.4 billion in 2025 and projected to grow at a compound annual growth rate (CAGR) of 5.8% during 2026–2034 to reach an estimated USD 5.7 billion by 2034, driven by increasing consumer awareness of health benefits and rising demand for exotic fruits across both mature and emerging economies (https://growthmarketreports.com/report/loquat-market). Despite this vigorous market growth, the corresponding market applications of loquat-derived products remain in an early stage of development. The current industrial chain is markedly fragmented: fruit pulp is processed largely as a food ingredient, leaves are utilized primarily for medicinal purposes, whereas peels and seeds are mostly discarded as waste (Duan et al., 2025; Carvalho et al., 2026). It should be emphasized that several of the downstream applications described in this review, including functional foods, nutraceuticals, oral-care products, skin-care ingredients, and biodegradable films, represent prospective or experimental directions supported by compositional and preclinical data rather than commercially established products (**Figure 9**). Wherever an application has already been demonstrated in practice or at pilot scale, this is stated explicitly in the text. This gap between the rapid expansion of the loquat market and the currently limited conversion of by-product tissues highlights the urgent need to establish the closed-loop, comprehensive circular industrial chain proposed above, thereby aligning industrial growth with the high-value and sustainable exploitation of loquat germplasm and metabolic resources.

**Figure 9:**
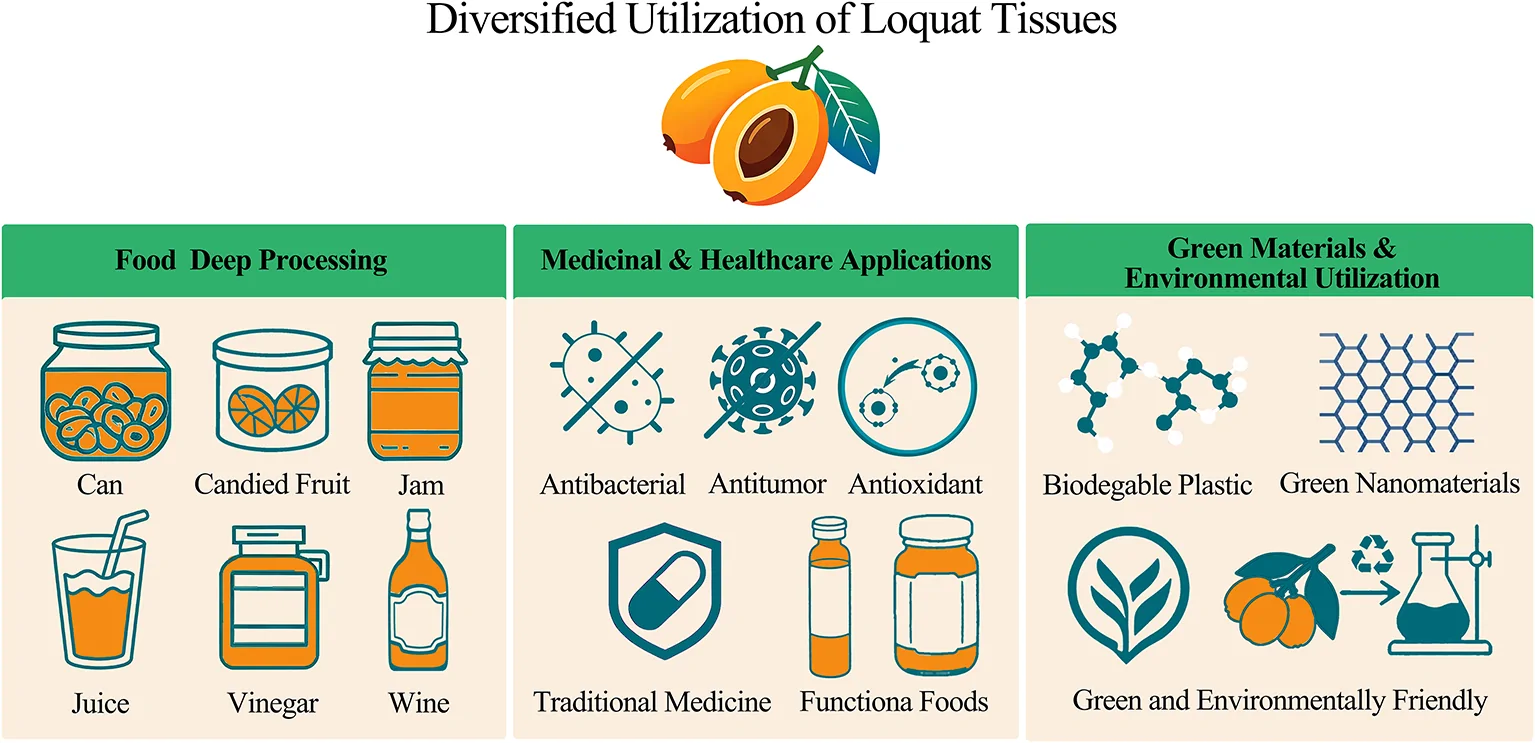
Overview concept map of diversified comprehensive utilization of various organs of loquat. This diagram illustrates the versatile potential of loquat biomass, primarily categorized into three major application directions. (1) Food Deep Processing: The edible portions of loquat can be processed into a wide variety of commercial products, including canned fruit, candied fruit, jam, juice, vinegar, and wine. (2) Medicinal & Healthcare Applications: Leveraging its intrinsic bioactive properties, loquat tissues are utilized for their antibacterial, antitumor, and antioxidant activities, contributing to the development of traditional medicines and functional foods. (3) Green Materials & Environmental Utilization: This direction highlights sustainable and ecological applications, focusing on the conversion of loquat-derived raw materials into biodegradable plastics and green nanomaterials, thereby promoting an environmentally friendly and circular bioeconomy approach. The results are derived from cell experiments or animal experiments and have not been verified by clear clinical data.

### 4.6 Limitations and heterogeneity of the reviewed literature

The studies compiled in this review are highly heterogeneous, which limits direct cross-study comparison. Variation arises from differences in loquat cultivars and germplasms, tissue developmental stages and harvest seasons, extraction solvents and procedures, analytical platforms (HPLC-UV, LC-MS, GC-MS, NMR) and quantification methods, and experimental models (chemical/enzymatic assays, cell lines, rodents) with inconsistent dose regimens and endpoints. Consequently, absolute metabolite contents and bioactivity magnitudes reported by different laboratories are not directly commensurable, and some findings on individual compounds or tissues are conflicting rather than convergent. Knowledge gaps include the scarcity of standardized, chemically defined extracts, the limited number of studies addressing compound interactions and multi-tissue comparisons, and the near-absence of clinical data. These limitations should be borne in mind when interpreting the quantitative comparisons and pharmacological summaries presented above.

A quantitative survey of the 763 English, peer-reviewed records retained in this review further substantiates the above caveats. Experimental studies predominate: 616 records (80.7%) involved animal (*in vivo*) models, 163 (21.4%) used cell-based (*in vitro*) assays, and only one record (0.1%) was a human randomized controlled trial—a placebo-controlled evaluation of loquat leaf extract on muscle strength and mass in healthy adults (Cho et al., 2016). In contrast, the many records mentioning “clinical” terminology upon keyword screening proved, on abstract-level verification, to be background statements, horticultural field designs, or methodological reports rather than human studies. The near-total absence of high-quality human clinical evidence means that the pharmacological activities summarized in this review—particularly the antioxidant, anti-inflammatory, and hypoglycemic claims—remain supported only by preclinical data, and translational conclusions should be drawn with caution.

Regarding the commercialization status of loquat-based functional products, the literature contains product-development and functional-evaluation studies—including a fermented tea prepared by tea-rolling of loquat leaves with hypotriacylglycerolemic and anti-obesity effects (Toshima et al., 2010), a functional beverage from loquat flower water extracts (Shen et al., 2023), a loquat flower tea derived from agricultural waste (Duan et al., 2025; Raza et al., 2025), dietary supplements of loquat leaf with validated HPLC quality-control methods (Xu et al., 2012; Seong et al., 2019), and value-added loquat fruit products (Raza et al., 2025)—but virtually no peer-reviewed report of a loquat-derived product that has been officially approved and commercially marketed as a functional food or health-care product. This disconnect between abundant preclinical efficacy data and the absence of marketed products highlights regulatory, safety-validation, and standardized-production barriers that remain to be overcome, and reinforces the industrial utilization gaps identified in Section 3.

### 4.7 Safety, decontamination and quality control in industrial processing

The food-medicine dual use of loquat tissues calls for integrated safety management across the entire industrial chain (Seong et al., 2019; Zhu et al., 2022). For seeds, the cyanogenic glycoside burden is the primary safety concern: hydrolysis of amygdalin liberates HCN in a dose-, tissue-, and processing-dependent manner, and seed-derived products must undergo validated detoxification procedures (e.g., controlled heating, soaking, fermentation, and kernel-coat removal) and comply with the HCN limits set by national food-safety standards before any edible or nutraceutical use (He et al., 2020). For fresh fruit and processed products, decontamination should address pesticide residues, heavy metals, and microbial contaminants through good agricultural practice (GAP), hazard analysis and critical control points (HACCP), and routine multi-residue monitoring. For medicinal preparations derived from leaves and flowers, quality control should follow pharmacopoeial specifications (e.g., China Pharmacopoeia) for marker-compound quantification, moisture, ash, and toxicological limits, and the approved new-food-raw-material status of loquat leaves and flowers should be accompanied by batch-to-batch chemical standardization. Establishing unified, evidence-based safety and quality-control standards is a prerequisite for the high-value and sustainable industrialization of loquat resources.

## 5 Conclusion

Current research on loquat has expanded from the edible pulp to the entire plant, yet most investigations remain concentrated on the traditionally used pulp and medicinal leaves. The tissue-specific surveys compiled herein show that triterpenoid acids, phenolic acids, and flavonoids are conserved across pulp, peel, seed, leaf, and flower, while each organ also accumulates signature compounds that underpin its particular pharmacological profile, including anti-inflammatory, antioxidant, hypoglycemic, and organ-protective activities. Fragmented research frameworks and discrete processing modes constitute the core bottlenecks hindering the high-quality upgrading of the loquat industry; emerging multi-omics profiling and synthetic biology strategies are resolving the molecular mechanisms of tissue-specific metabolic partitioning, and a full-industry-chain framework covering germplasm excavation, precise hierarchical processing, and targeted functional product exploitation is therefore technically feasible and industrially imperative. Full utilization of loquat’s tissue-specific metabolic differentiation traits, coupled with the integration of cutting-edge omics technologies and synthetic biology engineering, will facilitate the comprehensive high-value conversion of all loquat tissue resources and provide robust theoretical and technical support for the iterative upgrading of the food-medicine homologous loquat industry. To reflect the strength of the available evidence, three categories are distinguished: (i) established findings—tissue-specific metabolite distribution and antioxidant/anti-inflammatory activities documented *in vitro* and in animal models, together with current industrial processing practices; (ii) proposed mechanisms—WGD- and cold-signaling-based explanations of tissue-specific metabolism, which await direct experimental validation in loquat; and (iii) future applications—synthetic biology production, circular-economy valorization, and any implied therapeutic uses, none of which should be interpreted as clinically validated at present.
